# Trans‐Atlantic Dispersal and Introgression Explain Holarctic Disjunct Distributions in *Vanessa* Butterflies

**DOI:** 10.1111/mec.17781

**Published:** 2025-04-29

**Authors:** Aleix Palahí, Aurora García‐Berro, Vlad Dincă, Raluca Vodă, Leonardo Dapporto, Niclas Backström, Roger Vila, Naomi E. Pierce, Gerard Talavera

**Affiliations:** ^1^ Institut Botànic de Barcelona (IBB), CSIC‐CMCNB Barcelona Catalonia Spain; ^2^ Evolutionary Biology Program, Department of Ecology and Genetics (IEG) Uppsala University Uppsala Sweden; ^3^ “Grigore Antipa” National Museum of Natural History Bucharest Romania; ^4^ Naturéum – State Museum of Natural Sciences, Palais de Rumine Lausanne Switzerland; ^5^ ZEN Lab, Dipartimento di Biologia Università Degli Studi di Firenze Sesto Fiorentino Italy; ^6^ Institut de Biologia Evolutiva (CSIC‐Univ. Pompeu Fabra) Barcelona Catalonia Spain; ^7^ Department of Organismic and Evolutionary Biology, Museum of Comparative Zoology Harvard University Cambridge Massachusetts USA

**Keywords:** biogeographic disjunctions, demography, introgression, Last Glacial Maximum, long‐distance dispersal, *Vanessa* butterflies

## Abstract

Species with disjunct distributions have long puzzled evolutionary biologists and biogeographers. Long‐distance dispersal can play a pivotal role in generating intra‐specific disjunct distributions, initiating early stages of allopatric speciation and leading to eventual interspecific disjunctions. *Vanessa* butterflies exhibit diverse movement behaviours, from low‐dispersal species with restricted distributions to others that engage in annual extensive migratory cycles. The biogeographic history of *Vanessa* presents intriguing cases of both intra‐ and interspecific disjunctions. *Vanessa atalanta* is present in the Nearctic and Western Palearctic but is absent in Asia, while its sister species *V. tameamea* is endemic to Hawaii. *Vanessa indica* occurs only in Asia, and its sister species, *V. vulcania*, is endemic to Macaronesia. Here, we investigate this conundrum through population genomics and demographic analyses of *Vanessa atalanta* using ddRAD data from 70 samples across its entire distributional range, identifying two genetically differentiated populations separated by the Atlantic Ocean. Demographic simulations and phylogenetic analyses suggest that these originated via long‐distance dispersal from the Nearctic to Europe around the Last Glacial Maximum. Hybridisation tests revealed introgression between the Palearctic population of *V. atalanta* and *V. indica* , indicating that their distributions overlapped during *V. atalanta* 's colonisation of Europe. We hypothesise that *V. atalanta* caused a species displacement of *V. indica* from Europe to Asia, explaining their current allopatric distributions—a scenario that is supported by ecological niche modelling. Together, our results illustrate the role of long‐distance dispersal and species interactions in shaping complex biogeographic patterns.

## 1 Introduction

Extreme disjunct distributions, where related taxa occur in geographically distant, discontinuous regions and/or distinct biomes, represent the far end of the spectrum of allopatric distributions (Brown and Lomolino 1998). Within a species, such phylogeographic disjunctions can serve as templates for reproductive isolation, ultimately leading to allopatric speciation and, over time, the establishment of higher‐level taxa with disjunct biogeographic distributions (Mayr 1942; Coyne and Orr 2004).

Vicariance has traditionally been a dominant explanation for disjunct distributions, especially when divergence times align with the formation of physical barriers such as oceans, mountains, or deserts (Hennig 1966; Rosen 1978; Brown and Lomolino 1998). For example, the breakup of Gondwana partially shaped pantropical lineages (Sytsma et al. 2004; Barker et al. 2007; Renner et al. 2010) and Southern Hemisphere biodiversity (Sanmartín and Ronquist 2004), while the split of Laurasia explains numerous disjunctions in the Northern Hemisphere (Sanmartín et al. 2001). Transient land bridges, such as Beringia, also facilitated biogeographic connections, enabling dispersal and subsequent vicariant splits across continents (Sanmartín et al. 2001; Vila et al. 2011). However, the ancient nature of most vicariance events makes them unlikely drivers of recent disjunctions within species. Instead, long‐distance dispersal (LDD) offers a more plausible explanation for recent disjunctions, particularly when divergence times are inconsistent with geological events (Heaney 2007; Rincón‐Barrado et al. 2021). While ancient vicariant disjunctions are well documented among insects (Kim and Farrell 2015; Toussaint et al. 2017), growing evidence highlights a significant role of LDD in shaping disjunct distributions and contributing to lineage diversification, particularly among flight‐capable taxa (Lovejoy et al. 2006; Kodandaramaiah and Wahlberg 2007; Kergoat et al. 2012; Rota et al. 2016; Bourguignon et al. 2018; Torres‐Cambas et al. 2019; Kawahara et al. 2023; Suchan et al. 2024).

The consequences of establishing a disjunction for the taxa involved can vary widely, depending on the mechanism driving the disjunction. In scenarios of vicariance, where a population is split by the emergence of a geographic barrier, less immediate divergence in ecological niche is typically expected. This contrasts with taxa that disperse via LDD over a pre‐existing barrier, as these newly established populations are more likely to encounter different habitats and, consequently, distinct selective pressures (Rincón‐Barrado et al. 2021). Beyond the potential evolutionary consequences for the dispersing taxa, colonisation of new regions often impacts the receiving ecosystems through novel species interactions (Currat et al. 2008; Van der Putten 2012; Balik et al. 2023). These interactions can result in negative impacts for native species (Norberg et al. 2012), sometimes even leading to global extinction (Witte et al. 1992; Ricciardi et al. 2002). Less severe outcomes include ecological niche displacement (Race 1982; Kenward and Holm 1993) or competitive exclusion (Hingston and Wotherspoon 2017; Plentovich et al. 2018), which may still result in local extinctions or shifts in the geographic ranges of native species (Pyšek et al. 2017; Lebouvier et al. 2020). Such effects can be exacerbated by genetic introgression between non‐native and native species, as the introduction of maladaptive genes may disrupt locally adapted haplotypes or replace the original population through demographic or genetic swamping (Huxel 1999; Kidd et al. 2009; Fitzpatrick et al. 2010; Todesco et al. 2016; Popovic and Bernatchez 2021). Hence, understanding the demographic and phylogeographic history of species with disjunct distributions is crucial for explaining the emergence of discontinuities in spatial distributions of these taxa and for the broader ecological impacts on the communities with which they interact (Fryxell 1967).

In the Holarctic region, encompassing the Nearctic (North America) and the Palearctic (Europe and Asia), intercontinental disjunct distributions are abundant in plants, driven by both species dispersal and vicariance (Fryxell 1967; De‐Yuan 1983; Qian and Ricklefs 1999, 2000; Sanmartín et al. 2001). In animals, such patterns are less frequent and often associated with dispersal or relatively recent vicariance via Beringia, which facilitated disjunctions between the Nearctic and Eastern Palearctic regions (i.e., Asia) (Lafontaine and Wood 1988; Sanmartín et al. 2001). Insects exhibit a similar pattern, with inter‐continental disjunctions primarily observed in recently diverged taxa, aligning with periods of Beringian connectivity (Crowson 1981; Nordlander et al. 1996; Savage and Wheeler 1999; Gómez‐Zúrita 2004; Vila et al. 2011; Schär et al. 2018). If we exclude these nearly continuous Holarctic distributions of cold‐adapted taxa, within‐species intercontinental disjunctions across the Holarctic are exceedingly rare in insects, except in a few species with prominent migratory tendencies, such as the red admiral butterfly ( *Vanessa atalanta* ) and the monarch ( *Danaus plexippus* ), suggesting infrequent but possible trans‐oceanic dispersal (Pierce et al. 2014).

The butterfly genus *Vanessa* is a remarkable system for studying the origin of extreme disjunctions, given its cosmopolitan range and the prominent migratory nature of several species (Wahlberg and Rubinoff 2011). Migratory behaviour in *Vanessa* species ranges from obligate annual multigenerational movements, such as in *V. cardui* (Menchetti et al. 2019; Talavera et al. 2018, 2023; Reich et al. 2024), to geographically restricted endemics, such as *V. vulcania* in Macaronesia or *V. tameamea* in Hawaii (Wahlberg and Rubinoff 2011). Disjunctions in *Vanessa* occur at both inter‐ and intra‐specific scales. For example, the sister species *V. vulcania* (Macaronesia) and *V. indica* (East and Central Asia) display a striking geographical gap of > 7000 km over mostly contiguous land. *Vanessa atalanta* , on the other hand, presents an intriguing disjunct Holarctic distribution, occurring across the Western Palearctic (approximately west of the Urals) and much of the Nearctic (Mexico to Canada) but being absent from the Eastern Palearctic (Asia). In the Western Palearctic, *V. atalanta* undertakes regular multigenerational migrations, moving northward in spring and overwintering in the Mediterranean (Brattström, Bensch, et al. 2010; Brattström et al. 2018). Patterns of population structure globally and in the Nearctic remain unexplored, and prior studies within the Western Palearctic, using Amplified Fragment Length Polymorphism (AFLP) and mitochondrial DNA, report mito‐nuclear discordance (Brattström, Åkesson, et al. 2010; Dapporto et al. 2022), underscoring the need for comprehensive phylogeographic investigation. *V. atalanta* 's sister species, *V. tameamea* , is endemic to Hawaii, and the detailed biogeographic history behind their current distributions remains unclear (Wahlberg and Rubinoff 2011).

Here, we aim to investigate the role of long‐distance dispersal and interspecific interactions in shaping current disjunct distributions within *Vanessa* butterflies across the Holarctic. We pay particular attention to the role played by *V. atalanta* in this biogeographic conundrum, given its intriguing trans‐oceanic disjunction. First, we assess the demographic and phylogeographic history of *V. atalanta* , using extensive genetic sampling across the entire range. Second, we investigate the potential impact of *V. atalanta* 's range expansion on the recipient range by testing for genetic introgression between *V. atalanta* and five other *Vanessa* species occurring across the Holarctic, hypothesising a role of hybridisation and interspecific interactions in facilitating processes of species displacement (Currat et al. 2008). Finally, we incorporate ecological niche modelling (ENM) for both current and past climatic intervals to assess the role of niche evolution and historical connectivity in shaping the observed patterns.

All sources of evidence combined help to resolve long‐standing questions regarding the complex bio‐ and phylogeographic histories of *Vanessa* butterflies that led to the current Holarctic disjunctions. More broadly, our results highlight the importance of long‐distance dispersal and interspecific interactions in shaping species distributions.

## 2 Materials and Methods

### 2.1 Sampling

**FIGURE 1:**
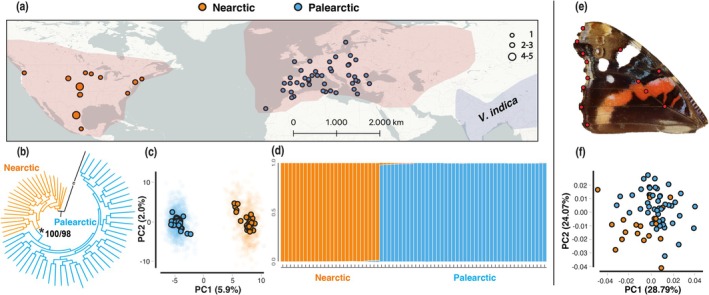
Sampling, morphometric and population genetic analysis in *V. atalanta*. (a) Sampling sites across the Nearctic and the Western Palearctic regions. Dot size indicates the number of individuals sampled in each site. The distribution of *V. atalanta* is indicated in light red and that of *V. indica* in light blue. (b) Phylogenetic tree for the 70 sequenced specimens of *V. atalanta*. Two *V. cardui* individuals are used as outgroups and collapsed into a single branch. The support for monophyly of the Palearctic samples is indicated with an asterisk, and the bootstrap support values are reported in the format SH‐aLRT/ultrafast‐bootstrap. (c) PCA obtained by subsampling one SNP per locus in the *V. atalanta* assembly. PC1 separates samples based on longitude of origin. Faded dots represent the PC coordinates for each sample in the 25 analyses using randomly subsampled SNPs. The highlighted dots represent the centroid coordinates for each individual. (d) The STRUCTURE plot for the 70 *V. atalanta* samples indicates a continental divide in population structure. (e) Thirteen fixed landmarks were used on the forewings of 65 *V. atalanta* individuals to test for differences in wing shape between continents. (f) A scatterplot of the two shape Principal Components resulting from geometric morphometric analysis. The variation explained by each component is indicated.

For phylogeographic assignments, we gathered molecular data from 70 samples of *Vanessa atalanta* (44 from Europe and Africa and 26 from North America) curated at the collections of the Institute of Evolutionary Biology (IBE, CSIC‐UPF, Spain), the Botanical Institute of Barcelona (IBB, CSIC, Spain), and the Museum of Comparative Zoology (Harvard University, USA) (Figure 1a, Table S1). Additionally, we sampled three individuals from each of five other *Vanessa* species occurring in the Palearctic and/or Nearctic: *V. cardui* (Palearctic), *V. cardui* (Nearctic), *V. anabella*, *V. virginiensis* , *V. indica* and *V. vulcania* (Table S1).

### 2.2 ddRAD Library Preparation and Sequencing

DNA was extracted from butterfly thoraces preserved in 100% ethanol and stored at −20°C. Approximately 10 mg of tissue was processed for each sample using the Omniprep DNA extraction kit (G‐Biosciences), following the manufacturer's protocol. Double‐digest restriction site‐associated DNA (ddRAD) genomic libraries were prepared for all individuals (70 *V. atalanta* and 18 from other *Vanessa* species) following the protocol of Peterson et al. (2012). EcoRI and BfaI (New England Biolabs) were used as restriction enzymes for genomic digestion through a 3‐h incubation step at 37°C, starting with a genomic DNA mass of at least 300 ng. Between 50 and 100 ng of digested DNA was used in a ligation reaction to add unique 5‐bp inline barcodes with a T4 DNA ligase (New England Biolabs). We used the 48 inline indices for EcoRI described in Peterson et al. (2012). Throughout the protocol, reaction cleanups were performed using Agencourt AMPure magnetic beads, and DNA was quantified using a Quant‐iT dsDNA HS Assay Kit (Thermo Fisher) in a Spectramax i3 plate reader (Molecular Devices) or a Qubit Fluorometer (Thermo Fisher). Fragments of 300 bp (280–320 bp) were selected from pools of 24–48 bp uniquely barcoded samples with a PippinPrep instrument and 2% agarose cassettes (Sage Science, Beverly, MA, USA). Fragment size distribution and concentration of the pools were measured with a Bioanalyzer instrument (Agilent Technologies). A final PCR step of 10 cycles was run to increase DNA amount and to add an Illumina P5 barcode using a Phusion High‐Fidelity PCR Kit (New England Biolabs). Libraries were finally pooled in equimolar amounts and sequenced in 150 bp paired‐end reads of the Illumina HiSeq2500 technology at the Harvard University Bauer Core Facility. Pools were spread across four Illumina lanes along with unrelated libraries, including 10% of PhiX RNA spike‐in to increase diversity.

### 2.3 ddRAD Data Processing and Assembly

Raw reads from the 70 *V. atalanta* individuals were demultiplexed and processed with *ipyrad* v0.9.81 (Eaton and Overcast 2020) using default parameters to optimise heterozygosity and error rates according to *ipyrad's* guidelines (https://ipyrad.readthedocs.io/en/latest/assembly_guidelines.html). Reads were mapped to the *V. atalanta* chromosome‐level genome assembly (Lohse et al. 2021). All loci that were present in less than 55 samples (< 20% of the total number of individuals) were filtered out. Finally, an all‐SNPs VCF file was generated. Sample statistics are available in Table S2.

### 2.4 Population Structure Analyses

Population structure in *V. atalanta* was first assessed through principal component analysis (PCA) as implemented in *ipyrad* v0.9.81 (Eaton and Overcast 2020). Twenty‐five independent replicates were conducted, each using a different randomly selected marker from each locus. The centroid for each sample was calculated based on these replicates.

To further assess admixture patterns, we used *STRUCTURE* v.2.3.4 (Pritchard et al. 2000), applying a filter to exclude sites with more than 10% missing data using *vcftools* v0.1.17 (Danecek et al. 2011). The numbers of lineages (K) tested ranged between 1 and 5. Ten independent runs were executed for each K, subsampling a random SNP per locus in each one. A total of 500,000 iterations were run, discarding the first 200,000 as burn‐in. *DeltaK* (Evanno et al. 2005) was used to evaluate the value for K that represented the best fit for the model. Based on the population assignment of the different individuals, nucleotide diversity (*π*) for each population and genetic differentiation (*F*_ST_) between clusters were calculated using *pixy* v.1.2.6 using –window_size 1 to account for the discontinuity of RAD‐seq data along the genome (Korunes and Samuk 2021).

The STRUCTURE analysis was repeated with the same parameters for each cluster identified during the first round to uncover potential extra levels of substructuring in the dataset.

### 2.5 Phylogenetics

*IQ‐TREE* v2.3.2 (Minh et al. 2020) was used to infer a maximum likelihood phylogeny for all 70 samples of *V. atalanta* , using two specimens of *V. cardui* as outgroups. *ipyrad* v.0.9.81 was used to obtain the assembly for all samples with the same parameters as previously used, and the resulting SNPs file was used as input for phylogenetic inference. IQ‐TREE was run with parameters ‐bnni ‐st DNA ‐m TEST. Branch support was tested with 10,000 ultra‐fast bootstrap replicates (with ‐bnni argument implementing an additional optimization step to correct for potential overestimation of branch support in case of model violations) and separately by 1000 replicates of the SH‐like approximate Likelihood Ratio Test (SH‐aLRT).

### 2.6 Demographic Inference With StairwayPlot2

The raw VCF file generated from the ddRAD assembly was filtered with *vcftools* v0.1.17 (Danecek et al. 2011) to remove markers with more than 20% missing data. We prioritised methods for demographic inference that rely on the site‐frequency spectrum (SFS) between populations, as these methods can accommodate the uneven distribution of ddRAD markers across the genome. Given the large size of our dataset (44 samples from the Palearctic and 26 from the Nearctic), subsampling a single marker per locus (to a maximum of 6250 markers; see Results) was insufficient to implement traditional SFS‐based demographic models. Therefore, we pruned the VCF file for linkage disequilibrium (LD) using *PLINK* v1.90 (Chang et al. 2015) with the command –indep‐pairwise 50 1 0.1. This step ensured that no pair of markers had an LD > 0.1, increasing the total number of SNPs available for SFS reconstruction and demographic modelling, while minimising a potential bias introduced by LD.

The LD‐pruned VCF file was first used to estimate the count of private alleles for each population using *bcftools* v1.11 (Danecek et al. 2021) with the –private flag. To ensure comparable sample sizes between populations, we performed five random subsamples of the Palearctic population, reducing it to 26 individuals (matching the sample size of the Nearctic population), and counted private alleles for each subsample. We then reconstructed the site‐frequency spectrum using *easySFS* (https://github.com/isaacovercast/easySFS; Gutenkunst et al. 2009). Initially, the tool was run with the –proj flag to determine the optimal projected sample sizes for each population. Subsequently, it was re‐run with *n* = 64 for the Palearctic and *n* = 38 for the Nearctic to generate a folded SFS for each population and a joint 2D‐SFS. To account for LD pruning, the number of monomorphic sites was adjusted proportionally to the retained SNPs.

The folded SFSs were then used to infer the demographic trajectories of each population during the Quaternary with *StairwayPlot2* v2.1.1 (Liu and Fu 2020) and identify key events shaping the demographic history of the species. We assumed four generations per year to convert generations before present (BP) into years BP and the down‐projected sample sizes from *easySFS*, along with a mutation rate of 2.9e‐9 per site per generation, based on another nymphalid, *Heliconius melpomene*, the only direct measure of mutation rate available for Lepidoptera to date (Keightley et al. 2015). All other parameters were set as default.

### 2.7 Model‐Based Demographic History Scenarios

To estimate the divergence time between the Nearctic and Palearctic demes of *V. atalanta* , identify the ancestral source population and infer migration patterns between demes that shaped the species' current distribution and genetic diversity, we developed and tested nine demographic models using *fastsimcoal2* v2.7 (Excoffier et al. 2021).

These models were designed to reconstruct the processes underlying the divergence of the two lineages from a shared ancestral population, integrating insights from *STRUCTURE* and *StairwayPlot2* analyses (Figure S1, Appendix S1). Specifically, the models incorporate two demes, reflecting the presence of two genetically distinct populations identified through population structure analyses. The timing and magnitude of demographic events inferred with *StairwayPlot2* were used to inform the prior distributions of the various parameter values (Figure S1, Appendix S1). The first three models were designed as “null models”, simulating an ancestral population that splits into two demes, with neither experiencing a bottleneck. These models differed by migration regime: no migration between demes after the split (M1), constant migration until the present day (M1‐mig), and a single‐migration event in each direction (M1‐one‐mig).

We subsequently tested two additional models that included a bottleneck in the Palearctic population: one with no migration (M2) and another with a single‐migration event (M2‐one‐mig). Similarly, two models were developed to explore a bottleneck in the Nearctic population: one without migration (M3) and one with a single‐migration event (M3‐one‐mig). Finally, we tested two models incorporating simultaneous bottlenecks in both populations: one with no migration (M4) and another with a single‐migration event (M4‐one‐mig).

Models that feature bottlenecks only in one population suggest directional migration, where the bottlenecked population represents the newly colonised region, characterised by a reduced population size relative to the source. We excluded models with constant migration rates, as trans‐Atlantic migration by *Vanessa* butterflies is extremely rare and unlikely to occur regularly. Graphical representations of all models, along with the parameters specific to each scenario, are provided in Figure S1.

The 95% CI values for the timing of events and population sizes obtained with *StairwayPlot2* were used as priors for the demographic models. A uniform distribution was set for all priors, except for a log‐uniform distribution for the migration rates between demes. In all cases, the simulations were allowed to explore parameter values outside of the prior distributions by not using the flag –bounded. The mutation rate was again set to 2.9e‐9 (Keightley et al. 2015). The .tpl and .est files for each tested model can be found in Appendix S1. The 2D‐SFS obtained from *easySFS* was used as input in all models.

One hundred repetitions of each model were run independently for *n* = 100,000 simulations and L = 50 ECM cycles. The run with the best fit (i.e., the smallest difference between MaxObsLhood and MaxEstLhood) for each model was selected, and different models were compared with the Akaike Information Criterion (AIC). A more restrictive penalty per parameter of 25 was used to avoid selecting a suboptimal model due to overparameterisation (Johri et al. 2021).

The mean and 95% CI values for each parameter of the best model were obtained by means of parametric bootstrap after simulating 50 2D‐SFS from the best‐fit model's .*par* file with *fastsimcoal2* (Excoffier et al. 2021) and later running the 50 replicates of the model again for each simulated SFS, with *n* = 50,000 and L = 30.

### 2.8 Introgression Tests

To assess evidence of introgression between *Vanessa* species, we used the *d3* statistic (Hahn and Hibbins 2019), a three‐sequence (one per taxon) test analogous to the ABBA‐BABA test (Patterson et al. 2012). This test estimates the extent of admixture by measuring the reduction in genetic distance resulting from introgression. In order to understand how common introgression is among *Vanessa* species across the Holarctic, we generated independent three‐sample assemblies for *V. atalanta* and each of its sympatric *Vanessa* species in the Nearctic ( *V. annabella* , *V. cardui* and *V. virginiensis* ) and in the Western Palearctic ( *V. cardui* and *V. vulcania*) with *ipyrad* v0.9.81 (Eaton and Overcast 2020) using default settings but allowing for no missing data. Additionally, we also tested for introgression between *V. atalanta* and *V. indica*, currently in allopatry, in order to test for potential historical contact. Three replicates of each test were performed, randomly selecting an individual from each of the taxa in each independent analysis (Table S3). For each replicate, a different three‐sample assembly was prepared. For introgression tests between Palearctic *V. atalanta* and *V. indica* , we used *V. atalanta* individuals from the westernmost part of its range (Spain/France/Greece; see Table S3), to minimise potential contemporary contact with *V. indica* . Genetic distances for all loci of the assemblies were calculated with the R package *ape* v5.6‐1 (Paradis and Schliep 2019) with the settings “methods = ‘percentage’, pairwise.deletion = TRUE, variance = FALSE”. *d3* was calculated based on the obtained distances, and the significance of the results was assessed by bootstrapping across all loci of the obtained assemblies.

### 2.9 Ecological Niche Modeling

To compare historical trends in the potential distributions of *V. atalanta* and *V. indica* , we built species distribution models and projected them at different time periods. Occurrence datasets for both species were retrieved from the Global Biodiversity Information Facility (GBIF 2020) using the R package rGBIF (Chamberlain and Boettiger 2017). The filtering options “capitals”, “centroids”, “equal”, “gbif”, “institutions”, “seas”, “zeros”, and “countries” were used in rGBIF to flag and remove potentially erroneous occurrences from the datasets. The R package spThin (Aiello‐Lammens et al. 2015) was used to spatially thin each species occurrence data by a minimum separation distance of 30 km between occurrences to minimise potential spatial biases in subsequent species distribution modelling. The locations of the resulting occurrences were visually inspected, and outliers far outside the known distributional range were excluded from the final datasets, which resulted in 1475 occurrences for *V. atalanta* and 144 occurrences for *V. indica* .

The environmental variables used for modelling were retrieved from the Paleoclim database (Brown et al. 2018) at 10 arc‐min (~20 km) resolution. From the 19 available bioclimatic variables, a subset was selected after excluding highly correlated variables with Pearson's coefficients higher than 0.8. The final subset of variables included mean diurnal temperature range (bio2), temperature seasonality (bio4), maximum temperature of warmest month (bio5) and precipitation of driest month (bio14).

The modelling area was set between −158° and 50° longitude and 14° and 70° latitude for *V. atalanta* and between 60° and 150° longitude and 4° and 65° latitude for *V. indica* to account for the extent of current distributions of these species. However, projections were extended in space to account for putative historical distributions, given the distributional shifts here studied: *V. atalanta* (−170° to 180° longitude; 1° to 74° latitude) and *V. indica* (−35° to 160° longitude; 1° to 74° latitude). The same set of variables was used for modelling the two species.

We used Biomod2 (Thuiller et al. 2024) to model the distribution of each species, using an ensemble approach. The modelling strategies used were Generalised Linear Models (GLM), Generalised Additive Models (GAM), Generalised Boosting Models (GBM), and Random Forests (RF). Occurrences were split in a proportion of 80%–20% for calibrating and evaluating the accuracy of the models, respectively. Each model was run five times, with the data randomly split in each run. A total of 500 pseudoabsences were set using the *sre* strategy, including five replicates of each pseudoabsence dataset. Ensemble models were obtained through weighted mean and committee averaging, and parameters for model ensemble were set to only account for runs with high evaluation scores of the true skill statistic (TSS) higher than 0.7 and the curve (ROC) higher than 0.8. The ensembled models were projected and plotted as probability rasters for four time periods: the Mid‐Pliocene warm period (ca. 3.205 Ma) (Hill 2015), the Last Interglacial (ca. 130 ka; Otto‐Bliesner et al. 2006), the Last Glacial Maximum (LGM, ca. 21 ka) (CHELSA v1.2, Karger et al. 2017), and the current period (1979–2013) (CHELSA v1.2, Karger et al. 2017).

### 2.10 Wing Morphometrics

Forewings of 65 *V. atalanta* individuals (Table S4) from both continents were photographed for comparative morphometric analyses. Samples were selected based on the integrity of their forewings and tpsDig v2.31 (Rohlf 2008) was used to score landmarks on the pictures of the wings' ventral side for all samples, corresponding to the junctions of the veins around the cells and along the termen (Figure 1e), as previously done in other butterfly species (e.g., Platania et al. 2020). Generalised Procrustes Analysis (GPA) was first applied to the landmark data to remove non‐shape variation in location, scale, and orientation, as well as to superimpose the objects in a common coordinate system (Rohlf and Slice 1990) and obtain Procrustes shape variables using the R package *geomorph* (Baken et al. 2021; Adams et al. 2023). The relative amount of shape variation attributable to the collection site (decimal latitude and longitude) of each specimen was tested by Procrustes ANOVA, using the procD.lm() function of the R package *geomorph* (Baken et al. 2021; Adams et al. 2023). The significance of the relationship was tested from the distributions obtained by resampling 999 permutations. This provided a linear model and estimates of the probability of this variation (“significance”) for a null model. Visual comparison of shape differences between Procrustes consensus configuration and shape at the extremes of regression lines was obtained by thin‐plate spline deformation plots using the function plotRefToTarget() of the R package *geomorph* (Baken et al. 2021; Adams et al. 2023).

## 3 Results

### 3.1 ddRAD Assembly and Variant Calling

The number of reads per sample in the raw data ranged between 818,674 and 1,968,127 (Table S2). This allowed for the assembly of 49,712 loci across the genome, of which 6250 were retained after filtering. The sequencing depth across RAD‐loci ranged between 10 and 15×. The total length of the assembly (sum of all concatenated RAD‐loci post‐filtering) was 1,949,477 bp, which indicates an average locus length of 312 bp. 113,344 SNPs were called in the raw assembly, of which 23,540 remained after LD pruning.

### 3.2 Inter‐Continental Population Structure in *V. atalanta*

The PCA revealed the existence of two distinct clusters of samples, corresponding to the populations occurring in North America and Europe/North Africa, respectively (Figure 1c). This differential clustering was consistent among independent replicate analyses, as both the centroid values for each sample and the random SNP subsampling showed no overlap between individuals from different continents (Figure 1c). The results of STRUCTURE (Pritchard et al. 2000) were consistent with the PCA, as *K* strongly indicated the presence of two distinct lineages in the data (Figure S2) corresponding to the Nearctic and Western Palearctic regions inhabited by *V. atalanta* (Figure 1d). No individuals of mixed ancestry were found, as all specimens could be attributed almost entirely (> 98%) to one lineage (Figure 1d). Combined, the results of both the PCA and STRUCTURE confirmed the existence of two well‐differentiated lineages in *V. atalanta* , one in the Nearctic region and the other in the Western Palearctic. The genetic differentiation between both populations (*F*_ST_) was 0.027.

Within‐continent population structure analyses suggested potential additional underlying levels of genetic structure, albeit without a clear geographic signal. In the Nearctic, *K* indicated the presence of three lineages (Figure S3b,c). While the PCA did not reveal distinct clustering (Figure S3a), specimens from Mexico and possibly New York exhibited slight differentiation. In the Palearctic, STRUCTURE identified two lineages (Figure S3e,f); however, the PCA similarly showed no geographical clustering (Figure S3d). In both cases, STRUCTURE failed to identify clear patterns that would allow for the reliable assignment of individuals to specific sub‐populations (Figure S3c,f).

The phylogenetic analysis provided strong support for a monophyletic group comprising all Western Palearctic samples, which were nested among the Nearctic samples (Figure 1b, Figure S4), supporting the hypothesis of a Nearctic origin for *V. atalanta* .

### 3.3 Demographic Oscillations Through the Quaternary

**FIGURE 2:**
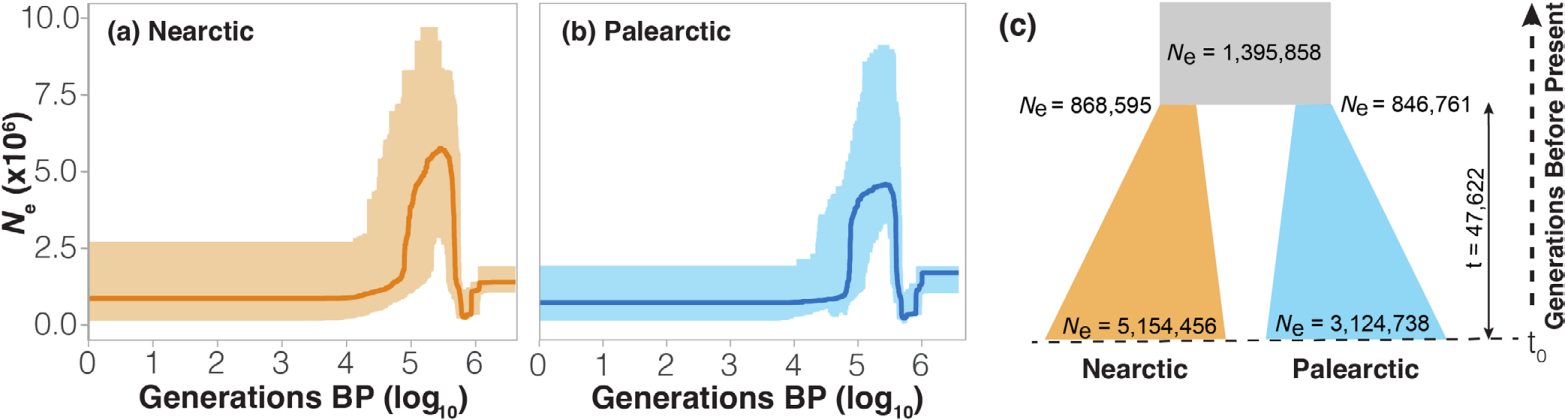
Demographic history of *V. atalanta*. (a) *StairwayPlot2* demographic trajectory for the Nearctic population. The solid lines indicate the median of 200 independent replicates, with the 95% CI represented by the shaded area. (b) *StairwayPlot2* demographic trajectories for the Palearctic population. (c) The demographic model (Model M4) that provided the best fit for the data. The orange sections depict demographic history in the Nearctic, where the species was originally restricted, while the blue section indicates the establishment of the Palearctic population of *V. atalanta* . An initial ancestral split resulted in a bottleneck for both demes, followed by a progressive increase in *N*_e_ to present‐day values. *N*_e_ is measured as the number of haploid genomes, migration rates express the proportion of migrating individuals per generation, and time is represented in generations before present (BP), with more recent times at the bottom of the axis.

*StairwayPlot2* (Liu and Fu 2020) was used to infer the oscillations of *N*_e_ during the Quaternary and to identify potential key events throughout the demographic history of the species. The two populations showed consistent trends (Figure 2a,b), both regarding the timing and the magnitude of *N*_e_ oscillations. Three different phases were identified: (i) a pronounced bottleneck ca. 600,000 generations before present (BP) – reaching *N*_e_ ≈ 200,000–250,000 – and posterior recovery to *N*_e_ > 4,000,000; (ii) a less pronounced *N*_e_ decline starting about 80,000 generations BP; and (iii) a stable phase of *N*_e_ during the last 10,000 generations (Figure 2a,b).

The inferred *N*_e_ with *StairwayPlot2* was overall higher for the Nearctic population in the period between bottlenecks (Figure 2a,b). Following the most recent bottleneck, the Nearctic population also had a higher effective population size (*N*_e_ = 874,175; 95% CIs: 137,941–2,723,161) compared to the Palearctic population (*N*_e_ = 730,305; 95% CIs: 133,231–1,932,873), indicative of a higher nucleotide diversity in the Nearctic population. In line with the demographic trajectories, the Nearctic population also showed a higher number of private alleles (*n* = 4576) than the five random subsamples of 26 Palearctic individuals (2879 < *n* < 2990), further supporting the higher genetic diversity harboured in the Nearctic population. These indirect estimations of the genetic diversity in each continent were confirmed by direct measurements of *π* (*π*_NA_ = 1.74e‐2; *π*_Pal_ = 1.65e‐2).

### 3.4 Model‐Based Inference of the Expansion of *V. atalanta*

All demographic models included two demes, according to the findings of the population structure analyses, and were provided with prior values according to the demographic curves inferred through *StairwayPlot2* (Figure S1, Appendix S1).

Model M4 (Figure 2c), which assumes no migration, provided the best fit to the 2D‐SFS, with a weight = 1 (Table S5). According to this model, an ancestral population (*n* = 1,395,858; 95% CIs: 1,391,633–1,400,082) experienced a split approximately 47,622 generations ago (95% CIs: 47,266–47,979). Both demes experienced a bottleneck following the split. In the Nearctic deme, *N*_e_ dropped to *n* = 868,595 (95% CIs: 838,529–898,660) after the bottleneck, followed by recovery to the current population size (*n* = 5,154,456; 95% CIs: 4,854,502–5,454,410). The Palearctic deme experienced a similar bottleneck (*n* = 846,761; 95% CIs: 819,263–874,259), but the current population size is smaller than that of the Nearctic (*n* = 3,124,738; 95% CIs: 3,024,979–3,224,497) (Figure 2c).

All other models showed poor support for the data (weights = 0–1.26e‐12) (Table S5), except for model M4‐single‐migration, which includes a single‐migration event between demes. Although this model provided a nearly identical likelihood to model M4, its additional parameters resulted in a higher AIC value and a lower weight (Table S5). This suggests that sporadic migration events across the Atlantic Ocean after the population split cannot be completely ruled out.

### 3.5 Evidence for Introgression Between Allopatric *V. atalanta* and *V. indica*

The three‐sequence assemblies obtained with *ipyrad* yielded between 853 and 2049 loci (Table S3). No significant evidence of introgression was found between *V. atalanta* and any of its sympatric species in the Nearctic (*V. cardui, V. annabella* and *V. virginiensis* ) or the continental Western Palearctic ( *V. cardui* ) (Figure 4b, Table S3). However, all three replicate tests between Palearctic *V. atalanta* and *V. vulcania*, which are sympatric in Macaronesia, revealed significant traces of introgression (d3 = 0.011–0.013, *p*‐value = 0.006–0.015; Table S3).

Most interestingly, evidence of hybridisation was detected between Palearctic *V. atalanta* and *V. indica* , which are currently allopatric (Figure 1a). All replicates of this test showed significant signals of introgression (d3 = 0.014–0.016, *p*‐value = 2.17e‐4–1.35e‐3; Table S3). In contrast, no introgression was detected between Nearctic *V. atalanta* samples and either *V. indica* or *V. vulcania*. Quantitatively, introgression also appeared more prevalent between allopatric Palearctic *V. atalanta* and *V. indica* (1.6% of the genome being introgressed) than between sympatric Palearctic *V. atalanta* and *V. vulcania* in Macaronesia (1.2% of the genome showing introgression).

The significant evidence of introgression between *V. atalanta* and *V. indica* in the Palearctic contrasts with their current allopatric distributions, indicating that they were sympatric in the past.

### 3.6 Ecological Niche Modeling

**FIGURE 3:**
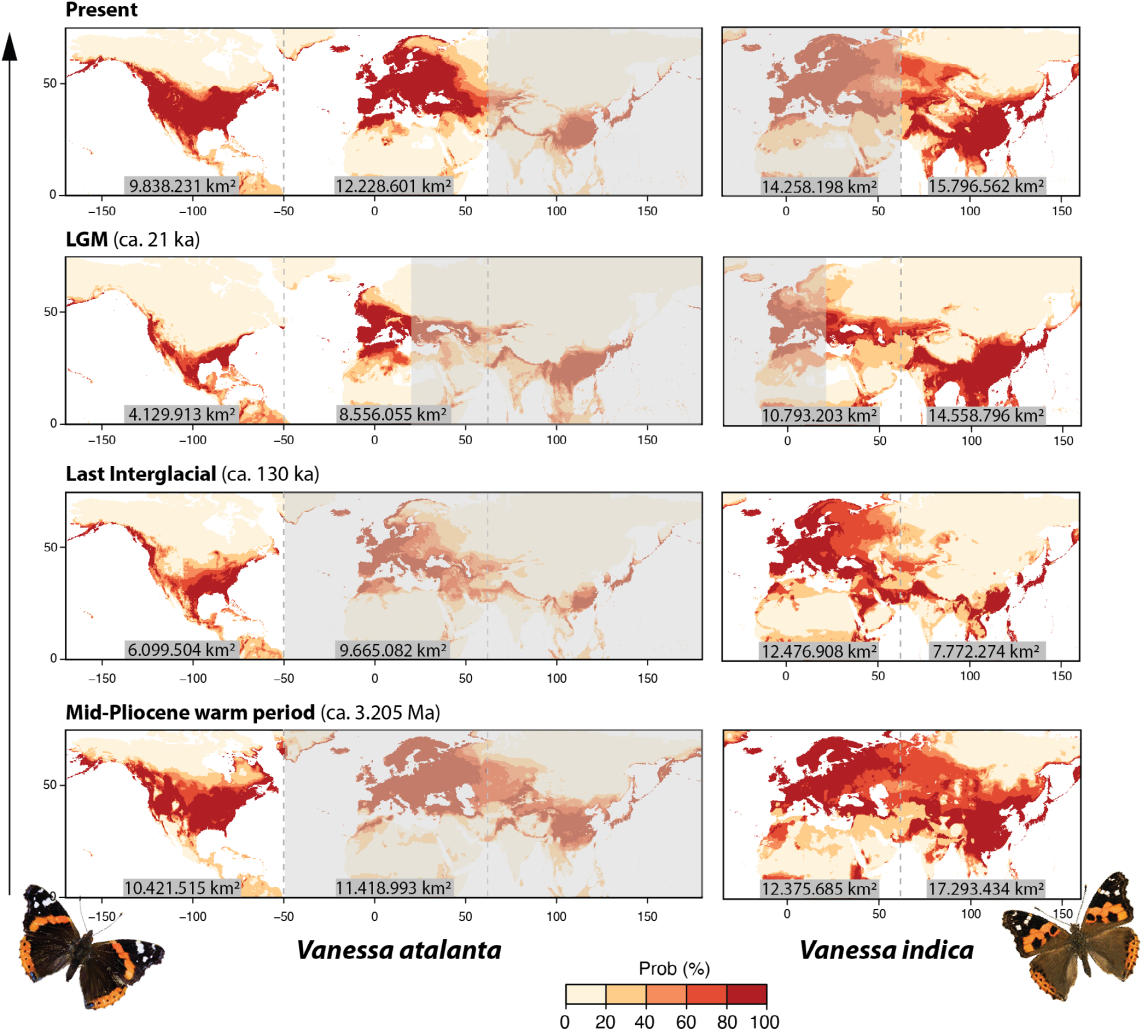
Species distributions models for *V. atalanta* and *V. indica* , with projections across time from the Mid‐Pliocene warm period (ca. 3.205 Ma), the Last Interglacial (ca. 130 Ka), the Last Glacial Maximum (ca. 21 Ka) and present times. Unshaded areas represent potentially occupied regions for each period. Suitable areas (in km^2^) for occurrence probabilities > 80% are indicated for the Nearctic, West Palearctic and East Palearctic regions. A red colour scale denotes occurrence probabilities from 0 to 100.

The historical distributions predicted through ENM offer a probabilistic framework for hypothesising the sequence of dispersal and interaction events that shaped current distributions of *Vanessa* butterflies across the Holarctic region. The projected models revealed several important trends (Figure 3): (i) Both the Nearctic and the Palearctic regions offered extensive suitable habitat for *V. atalanta* , while the entire Palearctic offered suitable habitat for *V. indica* since at least the mid‐Pliocene (ca. 3.205 Ma); (ii) the last interglacial period appears to have strongly influenced the distributions of both species. For *V. atalanta* , the suitable habitat in the Nearctic nearly halved (from ~10.4 M km^2^ to ~6.1 km^2^ when considering probabilities > 80%). For *V. indica* , there was a marked reduction in suitable area in the Eastern Palearctic and Oriental regions (from ~17.3 M km^2^ to ~7.8 M km^2^), while the suitable habitat in the Western Palearctic remained stable; (iii) the LGM caused a sharp drop in suitable area of *V. atalanta* in the Nearctic (~4.1 M km^2^), which was doubled in the Western Palearctic (~8.6 M km^2^). For *V. indica* , its potential distribution in the Western Palearctic remained high (~10.8 M km^2^), while it expanded substantially in the Oriental region and the Eastern Palearctic since the Last Interglacial (from ~7.8 M km^2^ to 14.6 M km^2^); (iv) in the present, the model aligns well with the known distributional ranges of *V. atalanta* in the Nearctic and Western Palearctic. Potential suitable habitat outside its current range remains restricted to the easternmost Palearctic, with minimal connectivity to the Western Palearctic. For *V. indica* , its known distribution is well represented in the Oriental and Eastern Palearctic regions. Projections to the Western Palearctic also show high suitability, comparable in area to its current range (~14.3 M km^2^ in the Western Palearctic vs. 15.8 M km^2^ in the Eastern Palearctic and Oriental regions), with strong geographic connectivity between Eastern and Western Palearctic regions.

### 3.7 Morphometrics

After GPA, the shape variation obtained using 13 landmarks on the forewings of *V. atalanta* showed a significant association with both longitude (Sum of Squares = 0.005, *R* ^2^ = 0.091, *F* = 6.476, *z* = 3.909, *p* = 0.001; Figure S5a) and latitude (Sum of Squares = 0.002, *R* ^2^ = 0.033, *F* = 2.309, *z* = 4.001, *p* = 0.029) in both the Palearctic and Nearctic regions. However, as also revealed by the low sum of squares values obtained for both variables, the overlap among samples from the Palearctic and the Nearctic was high (Figure 1f). The main tendency, as revealed by comparison of thin‐plate splines for extreme Eastern and Western specimens, is for more elongated wings in Nearctic specimens, although the difference is slight (Figure S5b,c).

## 4 Discussion

### 4.1 Population Structure Is Consistent With a Continental Divide in *V. atalanta*

The population genetic analyses, coupled with morphometric variation, provide compelling evidence of population structure in *V. atalanta* consistent with a continental divide across the Atlantic Ocean (Figure 1). Clustering analyses reveal a distinct lineage per continent (Figure 1c), with all individuals exclusively clustered within one of these lineages. No individuals with > 2% of their ancestry belonging to the alternative continental lineage were detected, emphasising the lack of admixture between these populations (Figure 1c). This sharp continental divide supports the demographic scenario suggested by the best fit model in which there is no secondary contact or migration between the two demes after the split of the original population (Figure 2c). Furthermore, this pattern is consistent with the phylogenetic findings that reveal a monophyletic clade of Palearctic individuals nested within the Nearctic individuals (Figure 1b, Figure S4). This provides additional support for the long‐term genetic isolation between the two populations, as well as the single origin of the Palearctic population from the Nearctic.

Our intracontinental STRUCTURE analyses for *V. atalanta* suggested mild but possible geographic clustering in the Nearctic. No geographic clustering was observed in the Western Palearctic despite STRUCTURE suggesting the presence of two distinct lineages (Figure S3), as previously observed with other genetic markers (Brattström, Åkesson, et al. 2010). This indicates that while genetic lineages exist, the annual, long‐distance migrations of *V. atalanta* may prevent the formation of strong geographic structuring. This scenario resembles the extensive latitudinal migrations of *Vanessa cardui* , which also exhibits a lack of genetic structure across its extensive Afro‐Palearctic range (Talavera and Vila 2017; García‐Berro et al. 2022; Talavera et al. 2023; Reich et al. 2025; Suchan et al. 2024). Similarly, it parallels the case of the monarch butterfly ( *Danaus plexippus* ) in North America, where no genetic differentiation is observed between eastern and western populations, despite their distinct behaviours in the choice of migratory routes (Talla et al. 2020).

In agreement with the genetic differentiation found between continents, the morphometric analysis also revealed slight differences in wing shape between *V. atalanta* specimens from both populations (Figure S5), although these differences are not sufficiently great to enable sample origin to be assigned based on this morphological trait alone. Such differences may be the result of adaptations to different flight strategies in regions that may differ in wind or other abiotic characteristics (Betts and Wootton 1988; Le Roy et al. 2021; Chan et al. 2023). Overall, the classification of *V. atalanta* in two subspecies—*V. atalanta atalanta* (Linnaeus 1758) in the Palearctic and *V. atalanta rubria* (Fruhstorfer 1909) in the Nearctic—seems to be justified.

### 4.2 Phylogeographic Disjunction as a Consequence of a Trans‐Atlantic Dispersal Event

The best‐supported model for *V. atalanta* suggests a demographic history characterised by an ancestral population splitting, with both resulting demes experiencing a bottleneck as a result, followed by a progressive recovery of *N*_e_ (Figure 2c). This ancestral population experienced a pronounced bottleneck prior to splitting, evidenced by the shared demographic trajectories of both populations in *StairwayPlot2* (Figure 2a,b). To translate these demographic events into specific timepoints, it is crucial to have an accurate estimate of the voltinism of the species. Given that voltinism is an environmentally dependent plastic trait that may have changed over time (Bale et al. 2002; Tobin et al. 2008; Altermatt 2010), a conservative range of 2–4 generations per year may be considered plausible in *V. atalanta* .

With 2–4 generations per year, the ancestral bottleneck perceived in *StairwayPlot2* (ca. 600,000 generations BP; Figure 2a,b) coincides with the cyclic oscillations in temperature and ice sheet coverage that characterised the intercalating interglacial and glacial periods of the Pleistocene in the Holarctic region (Williams et al. 1998). With four generations per year (ca. 150 kya), the bottleneck would align with the Penultimate Glacial Period (MIS 6; 190–130 kya), while bivoltinism (ca. 300 kya) places it at the beginning of an older glacial period (MIS 8; 300–240 kya) (EPICA Community Members 2004). Throughout the Northern Hemisphere, cold glacial periods forced many species to retreat towards southern refugia (Hewitt 1996, 2000; Taberlet et al. 1998; Dapporto et al. 2019, 2024), with restrictions in geographic range potentially leading to declines in genetic diversity (Hewitt 1996; Arenas et al. 2012; Rogan et al. 2023). This pattern has been widely documented across multiple taxa (Beck et al. 2008; Pușcaș et al. 2008; Conord et al. 2012), including arthropods (Gratton et al. 2008; Dapporto 2009; Dapporto et al. 2012, 2024; Bidegaray‐Batista et al. 2016; Maresova et al. 2021; Cao et al. 2022).

A projection of our ecological niche model to the LGM (ca. 21 Ka) highlights the impact of glacial periods on the species' demographic history. The model suggests that *V. atalanta* likely experienced a significant contraction in its potential distributional range during glacial times compared to warmer periods such as the Last Interglacial (ca. 130 Kya) or the present day (Figure 3), aligning with the demographic trajectories inferred from genetic data (Figure 2a,b). Interestingly, the model also predicts a smaller range for *V. atalanta* during the Last Interglacial compared to the current interglacial. The Last Interglacial, which was warmer than the present (Otto‐Bliesner et al. 2013; Bakker et al. 2013), may have driven *V. atalanta* out of its climatic niche in several regions (Figure 3). Similar responses are observed in insects in the current context of increasing temperatures (Wilson et al. 2005; Rubenstein et al. 2023). Overall, the dramatic climatic fluctuations of the Pleistocene, including glacial and interglacial cycles, are likely associated with the demographic bottleneck detected over that period.

The LGM appears to be a period of critical importance in the demographic history of *V. atalanta*, as the population split between the Nearctic and the Palearctic took place ca. 48,000 generations BP (Figure 2c), coinciding with the end of the Last Glacial Period (ca. 115–10 kya) under the assumption of 2–4 annual generations (ca. 12–24 kya). In a bivoltine scenario, the split aligns with the time of maximum ice sheet coverage in the Northern Hemisphere, around 21 kya (Bard et al. 1990; Armstrong et al. 2019). The recency of this divergence between continental lineages rules out vicariance as a plausible explanation for the phylogeographic disjunction. It also discards the possibility of a range expansion resulting from a recent human‐mediated introduction, as has been documented in other Lepidoptera such as *Pieris rapae* (Ryan et al. 2019), *Thymelicus lineola* (D'Ercole et al. 2022), and *Noctua pronuba* (Lentz 2006). Instead, this unambiguously supports an LDD event as the cause of the intercontinental divergence.

This LDD event might have resulted either from a trans‐Beringian or a trans‐Atlantic dispersal. Beringia has often served as a dispersal corridor between the Nearctic and Palearctic for various insect species (Vila et al. 2011; Rota et al. 2016; Schär et al. 2018; Kohli et al. 2021; Maresova et al. 2019). Similarly, cases of trans‐oceanic dispersal have also been verified (Lovejoy et al. 2006; Gillespie et al. 2012; Lalonde and Marcus 2022; Suchan et al. 2024). However, for *V. atalanta* , a range expansion across Beringia appears unlikely due to its absence in the Eastern Palearctic. Our ecological niche models further support this, showing a historical lack of suitable habitat in Northeast Asia for *V. atalanta* (Figure 3). Instead, the evidence strongly points to a trans‐Atlantic dispersal. The ecological niche models reveal an important contraction of *V. atalanta* 's suitable range during the LGM in both the Palearctic and the Nearctic (Figure 3). Moreover, from the Last Interglacial through the Last Glacial Period, much of *V. atalanta* 's suitable range in North America was concentrated along the eastern coast (Figure 3). This region would have been a key location for initiating a trans‐Atlantic dispersal event. The environmental pressures of the LGM may have increased the frequency of LDD events, facilitating successful trans‐Atlantic dispersal between the Nearctic and the Palearctic.

**FIGURE 4:**
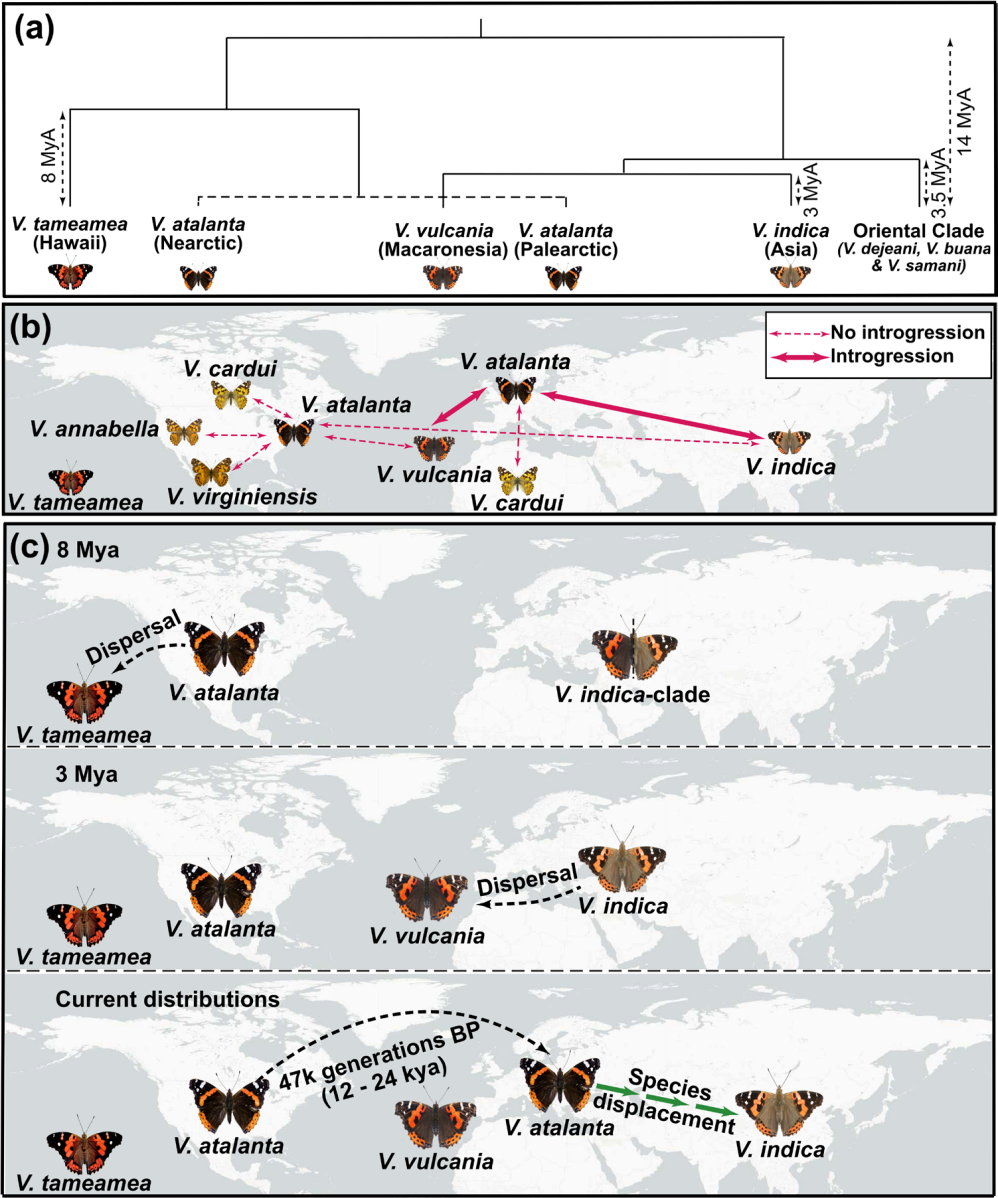
Dispersal and introgression explain the biogeographic history of disjunctions in *Vanessa* butterflies. (a) Phylogeny of *Vanessa* butterflies, highlighting disjunct distributions within the genus (adapted from Wahlberg and Rubinoff 2011). (b) Evidence of introgression among *Vanessa* species in the Northern Hemisphere. Fine dashed lines represent all introgression tests performed between *V. atalanta* and other *Vanessa* species in the Holarctic, except for *V. tameamea* . Thick solid lines indicate significant introgression between species. (c) Proposed sequence of dispersal events and species interactions that led to the current disjunct distributions in *V. atalanta* populations, as well as between the sister species *V. indica* and *V. vulcania*.

Our best demographic model did not explicitly determine the continent of origin for *V. atalanta* or the direction of the LDD. However, multiple lines of evidence suggest a Nearctic origin of the species. Biogeographically, an origin in the Nearctic is the most parsimonious hypothesis, supported by the fact that *V. tameamea*, the sister species of *V. atalanta* (divergence time ca. 8 Mya; Figure 4), is endemic to Hawaii (Wahlberg and Rubinoff 2011) and that a colonisation event of the archipelago most likely happened from North America. This scenario does not contradict a possible Asian origin of the *V. atalanta* species group (Wahlberg and Rubinoff 2011), as the common ancestor of both species could have dispersed to the Nearctic before the divergence of *V. atalanta* and *V. tameamea*. This hypothesis is more realistic than an alternative scenario in which their common ancestor originated in Asia and independently expanded to Hawaii, North America, and Europe, only to later go extinct in the original Eastern Palearctic range.

Secondly, the demographic model suggests that the current effective population size (*N*_e_) of the Nearctic populations is significantly larger than that of the Western Palearctic (Figure 2c). These indirect estimates of genetic diversity are consistent with the higher nucleotide diversity (*π*) and greater number of private alleles observed in the Nearctic population. Together, these observations indicate that the Nearctic deme of *V. atalanta* harbours more genetic variation, a characteristic commonly associated with source populations (Nei et al. 1975). Upon establishment, it is likely that only a subset of the genetic variation was introduced to the Palearctic as a result of LDD.

Thirdly, the phylogenetic analysis shows that all Western Palearctic samples form a monophyletic group nested within the Nearctic lineage (Figure 1b). This further reinforces the evidence that the Nearctic population is the source, with the Western Palearctic deme originating from dispersal events during a limited timeframe.

Finally, during the time of the split (approximately during the LGM), prevailing wind trajectories across the Atlantic were predominantly eastward, making westward dispersal less likely. Wind patterns are known to facilitate LDD events, increasing the likelihood of insects reaching new land masses across vast water bodies (Gillespie et al. 2012; Kling and Ackerly 2020; Suchan et al. 2024). Many flying animals, including birds (Gillespie et al. 2012; Shamoun‐Baranes et al. 2017) and insects (Howard and David 1991; Rosenberg and Burt 1999; Lovejoy et al. 2006; Lorenz 2009; Reynolds et al. 2016; Suchan et al. 2024), benefit from these wind‐assisted LDD events. Several insects, such as the butterflies *Danaus plexippus* or *Vanessa virginiensis* , are thought to have crossed the Atlantic from North America to Europe in relatively recent times and established permanent populations (García‐Barros et al. 2004; Fernández‐Haeger and Jordano Barbudo 2009; Pierce et al. 2014; García et al. 2015). During the LGM, the polar front jet stream, which provided strong eastward winds over the Atlantic, was much stronger than today (Luetscher et al. 2015; Wang et al. 2018). This intensified jet stream also extended into lower latitudes, even reaching the Mediterranean (Rind 2009), overall creating a plausible scenario for a successful dispersal of *V. atalanta* .

Upon colonising the Western Palearctic during the LGM, *V. atalanta* likely encountered ample suitable habitat, facilitating a steady colonisation. According to the ecological niche model, its potential suitable range in the Western Palearctic could have been up to twice the size of that in the Nearctic (Figure 3). Its primary host plant, *Urtica dioica* , persisted throughout the LGM as far north as the British Isles (Taylor 2009), and thus it was likely present in the Mediterranean region even in greater numbers due to favourable climatic conditions, providing the resources needed to establish and thrive.

At the time of the split, the demographic model indicates similar *N*_e_ (despite slightly larger in the Nearctic) in both diverging demes. Typically, range expansions are associated with very low genetic diversity in newly established populations, so how is it possible that a group of immigrants arriving from across the Atlantic Ocean did not cause a bottleneck? It is theoretically possible for a relatively small number of founders to establish a new population without a considerable reduction in *N*_e_ (Nei et al. 1975), provided that they carry sufficiently high levels of genetic diversity. In *V. atalanta*, our measurements of genetic diversity, although slightly higher than previous estimates (Mackintosh et al. 2019; Lohse et al. 2021), are relatively low given the positive association between genomic heterozygosity and migratory behaviour observed in butterflies (García‐Berro et al. 2022). Still, and despite the fact that the best model does not favour a scenario of recurrent post‐split migration, it seems implausible that a single LDD event involving a reduced number of individuals could have introduced enough genetic diversity from the source into Europe to avoid experiencing genetic erosion. Our best model is a close competitor of the M4‐one‐migration model (Figure S1), which includes an additional migration event after the split, showing almost identical likelihoods but lower weight due to a higher number of parameters (Table S5). This further suggests that some sporadic migration events could have taken place after the split of both populations. The strength and convenient southward shift of the polar jet stream during the LGM could have facilitated multiple cohorts of individuals arriving in the Palearctic from the Nearctic, during a relatively short period of time, introducing sufficient genetic diversity to mask a bottleneck in population size (Rind 2009; Luetscher et al. 2015).

### 4.3 Introgression by *V. atalanta* in the Palearctic Explains Biogeographic Disjunction Between *V. indica* and *V. vulcania*

We found significant evidence of genetic admixture between the Palearctic population of *V. atalanta* and *V. indica* . In contrast, no hybridisation was detected between Nearctic *V. atalanta* and *V. indica*, nor between *V. atalanta* and any sympatric congenerics, except for its Palearctic populations and *V. vulcania* (Figure 3b, Table S3). Finding introgressive hybridisation between *V. atalanta* and *V. vulcania* is not entirely surprising, given their co‐existence in Macaronesia and their relatively recent divergence time (ca. 12 Myr), compared to the more distantly related sympatric species that *V. atalanta* encounters in the Nearctic, which fall outside the *V. atalanta* species group (Wahlberg and Rubinoff 2011). The case of *V. atalanta* and *V. indica* , however, is particularly intriguing since these species are currently allopatric. While rare dispersal events (not yet documented) cannot be entirely ruled out as a partial explanation for the introgression signal, we minimised this possibility by selecting the most geographically distant samples from our dataset (Table S3). The observed introgression suggests a historical period of sympatry between these species. We hypothesise that *V. atalanta* and *V. indica* co‐existed when *V. atalanta* first established in the Western Palearctic, but that, over time, *V. atalanta* subsequently displaced *V. indica* , contracting its range and leading to their current allopatric distribution (Figure 4).

Modelled historical distributions of *V. indica* provide further evidence of the possibility of this overlap, as they indicate ample climatically suitable areas in the Western Palearctic over history (Figure 3). Interestingly, its suitable range was minimal in the Oriental and Eastern Palearctic regions during the Last Interglacial, suggesting that the majority of its population could have concentrated in the Western Palearctic. In contrast, after the Last Interglacial and leading up to the LGM, *V. indica* 's suitable range rapidly expanded in the Oriental and Eastern Palearctic regions, while it contracted in the West Palearctic. This contraction coincided with *V. atalanta* 's colonisation of the Western Palearctic, creating opportunities for interaction between the two species, as demonstrated by their genetic introgression. These interactions, coupled with environmental pressures associated with the LGM, likely contributed to *V. indica* 's eastward retreat. This displacement to the Eastern Palearctic ultimately resulted in the current wide distributional gap between *V. indica* and its Macaronesian sister species, *V. vulcania* (Figure 1a).

In addition to environmental factors, the displacement of *V. indica* in the Western Palearctic may have been driven by multiple biotic factors, including host plant availability and parasitoid pressure. *V. atalanta* , which primarily uses *Parietaria* spp. and *Urtica dioica* as host plants, would have encountered an abundance of these plants in the Western Palearctic. In contrast, *V. indica* 's main host plants in Asia, *Girardinia diversifolia* and *Boehmeria nivea* (Gawande et al. 2014), are currently absent in the region. Notably, these two plant species belong to the same family, Urticaceae, and *V. indica* has been shown to use *Urtica* spp. as hostplants (Wynter‐Blyth 1957, Janz and Nylin 1997, Kunte and Sengupta 2025). It is therefore reasonable to speculate that both butterfly species might have competed for the host plants currently used by *V. atalanta* . Without direct competition early on, *V. indica* may have initially exploited an ecological niche in the Western Palearctic that *V. atalanta* outcompeted upon its establishment during the LGM. Additionally, it is reasonable to consider that the absence of specialised parasitoids against *V. atalanta* and possible new diseases it might have introduced may have disproportionately impacted *V. indica* and hastened its displacement (Bennett 1993; Feener 1981; Hanks et al. 1995). Furthermore, the combination of a large effective population size (*N*_e_) in *V. atalanta* and its hybridisation with *V. indica* could have played a role in the displacement. Through hybridisation, *V. atalanta* may have acquired adaptive alleles or haplotypes from *V. indica* that enhanced its competitive edge, as previously demonstrated in a wide array of species (Seefeldt et al. 1998; Choler et al. 2004; Song et al. 2011; Stelkens et al. 2014; Pfennig et al. 2016; Pierce et al. 2017).

The sequence of events here described (Figure 4) is consistent with the phylogeny of the *Vanessa* genus (Wahlberg and Rubinoff 2011), but it does not support a proposed American origin of the *V. indica* ‐clade, based on the placement of the Nearctic fossil *V. amerindica* within the *V. indica* ‐clade (Vane‐Wright 2007), a claim that was later challenged by De Jong (2017). The current distributions of *V. indica* and *V. vulcania* are more consistent with a common ancestor in the Western Palearctic, where *V. indica* remained widespread across the mainland, while *V. vulcania* speciated in Macaronesia long before the establishment of a new population of *V. atalanta* of Nearctic origin.

In summary, our study shows how the onset of a phylogeographic disjunction within a species triggered new species interactions, ultimately leading to a biogeographic disjunction between two other sister species. *Vanessa* butterflies offer a compelling case study illustrating how the exploration of a species' phylogeography, coupled with evidence of interactions between closely related species, can elucidate current disjunct distributions within a clade. More broadly, our findings demonstrate the synergy between the study of intra‐ and interspecific processes and their combined utility in efforts to understand modern biogeographical patterns.

## Author Contributions

A.P. and G.T. conceived the study. V.D., R.Vo., L.D., R.Vi., and G.T. collected samples. G.T. carried out laboratory work. A.P., A.G.‐B., L.D., and G.T. analysed data. R.Vi., N.E.P., and G.T. secured funding and laboratory facilities. A.P. and G.T. wrote the first version of the manuscript. All authors contributed to interpreting results and edited and approved the final version of the manuscript.

## Conflicts of Interest

The authors declare no conflicts of interest.

## Supporting information


Appendix S1

## Data Availability

Sequencing reads have been deposited in the ENA (European Nucleotide Archive) database under accession number PRJEB71476.
