# Supplementary material for: Trans‐Atlantic Dispersal and Introgression Explain Holarctic Disjunct Distributions in Vanessa Butterflies
Source: Mol Ecol. 2025 Apr 29;34(11):e17781. doi: 10.1111/mec.17781 (PMC12100595; doi:10.1111/mec.17781)
Supplement: Supplementary file 1 — Appendix S1 [file MEC-34-e17781-s001.pdf]

MOLECULAR ECOLOGY

Supplemental Information for:

Transatlantic dispersal and introgression explain Holarctic disjunct distributions in *Vanessa* butterflies

Aleix Palahí, Aurora García-Berro, Vlad Dincă, Raluca Vodă, Leonardo Dapporto, Niclas Backström, Roger Vila, Naomi E. Pierce, Gerard Talavera

Table of Contents:

**Supplementary Table 1**	Page 2
**Supplementary Table 2**	Page 4
**Supplementary Table 3**	Page 6
**Supplementary Table 4**	Page 7
**Supplementary Table 5**	Page 9
**Supplementary Figure 1**	Page 10
**Supplementary Figure 2**	Page 11
**Supplementary Figure 3**	Page 12
**Supplementary Figure 4**	Page 13
**Supplementary Figure 5**	Page 14
**Appendix 1**	Page 15

Supplementary Table 1. Sampling coordinates of the sequenced *V. atalanta* individuals and other species across the Holarctic.

Sample ID	Country/State	Region	Latitude	Longitude
04G134	Turkey	West Palearctic	40.168	29.095
08H295	Spain	West Palearctic	41.634	2.353
08H389	Spain	West Palearctic	39.535	-0.459
08H490	Spain	West Palearctic	40.643	-2.813
08H640	Spain	West Palearctic	36.199	-5.330
08H927	Spain	West Palearctic	40.306	-5.806
08L489	Spain	West Palearctic	42.765	0.711
08M443	Romania	West Palearctic	44.062	27.649
08P687	Spain	West Palearctic	42.949	-5.744
08R278	Spain	West Palearctic	42.167	2.364
08R323	Spain	West Palearctic	39.732	2.648
09X874	Italy	West Palearctic	40.049	9.300
10B686	France	West Palearctic	44.032	6.172
11D114	Italy	West Palearctic	36.785	11.981
11E103	Malta	West Palearctic	35.859	14.400
11F719	Morocco	West Palearctic	34.089	-4.183
11F924	Morocco	West Palearctic	33.542	-5.115
12L581	Portugal	West Palearctic	37.025	-8.939
12M102	Greece	West Palearctic	35.310	23.810
12N243	Tunisia	West Palearctic	36.294	9.811
12P542	France	West Palearctic	44.013	3.849
13T444	Tunisia	West Palearctic	36.726	8.705
13U246	Italy	West Palearctic	46.193	12.351
14C075	Sweden	West Palearctic	59.749	18.656
14C290	Greece	West Palearctic	41.293	24.054
14C924	Bulgaria	West Palearctic	41.764	23.156
14D218	Austria	West Palearctic	47.283	11.884
14E990	Serbia	West Palearctic	44.122	20.015
14F318	Serbia	West Palearctic	43.392	22.612
14G912	Greece	West Palearctic	40.320	21.649
14H461	Greece	West Palearctic	37.527	22.346
14I786	Slovakia	West Palearctic	49.239	20.101
14V235	Belgium	West Palearctic	50.741	2.875
14W965	UK	West Palearctic	50.570	-3.900
15B183	Minnesota	Nearctic	44.780	-93.384
15B535	Oklahoma	Nearctic	34.744	-98.532
15B536	Oklahoma	Nearctic	34.744	-98.532
15B589	Kansas	Nearctic	38.899	-98.520
15B590	Kansas	Nearctic	38.899	-98.520
15B591	Kansas	Nearctic	38.899	-98.520
15B598	Kansas	Nearctic	38.899	-98.520
15B641	Oregon	Nearctic	45.255	-122.904
15B667	Nebraska	Nearctic	42.684	-103.518
15B668	Nebraska	Nearctic	42.684	-103.518
15B682	Minnesota	Nearctic	45.283	-95.967
15B732	Wisconsin	Nearctic	43.092	-89.336
15B802	New York	Nearctic	40.768	-73.974
15B803	New York	Nearctic	40.731	-74.000

15B804	New York	Nearctic	40.731	-74.000
15B805	New York	Nearctic	40.697	-73.999
15B806	New York	Nearctic	40.719	-74.016
15B811	Massachussets	Nearctic	42.374	-71.117
15F232	Portugal	West Palearctic	40.370	-8.362
15F862	France	West Palearctic	46.433	-1.027
15L546	Italy	West Palearctic	43.993	8.153
16B298	France	West Palearctic	45.920	5.392
16B877	Nuevo Leon	Nearctic	25.554	-100.268
16B888	Nuevo Leon	Nearctic	25.154	-99.826
16B889	Nuevo Leon	Nearctic	25.154	-99.826
16B891	Nuevo Leon	Nearctic	25.154	-99.826
16B892	Nuevo Leon	Nearctic	25.154	-99.826
16E411	Puebla	Nearctic	18.829	-97.811
16E439	Mexico DF	Nearctic	-	-
16E491	North Carolina	Nearctic	35.639	-77.361
16E548	Germany	West Palearctic	50.491	11.556
16I034	Poland	West Palearctic	52.819	14.235
16I365	Poland	West Palearctic	51.406	23.124
16I757	Poland	West Palearctic	53.455	20.736
16I905	Slovakia	West Palearctic	48.900	19.738
16L312	Spain	West Palearctic	28.391	-16.562

16E165	China (*V. indica)*	East Palearctic	40.966487	114.987038
16E164	China (*V. indica)*	East Palearctic	40.966487	114.987038
16E178	Vietnam (*V. indica)*	East Palearctic	-	-
16L310	Spain (*V. vulcania*)	Macaronesia	28.390996	-16.561939
16L311	Spain (*V. vulcania*)	Macaronesia	28.390996	-16.561939
16L310	Spain (*V. vulcania*)	Macaronesia	28.390996	-16.561939
09X202	France (*V. cardui*)	West Palearctic	43.896000	5.920000
09V399	Spain (*V. cardui*)	West Palearctic	40.650000	0.761000
14H393	Greece (*V. cardui*)	West Palearctic	38.476900	22.596600
16E483	US (*V. virginiensis*)	Nearctic	35.638826	-77.360627
15B537	US (*V. virginiensis*)	Nearctic	34.744115	-98.531672
16B895	US (*V. virginiensis*)	Nearctic	25.154425	-99.825984
15B724	US (*V. cardui*)	Nearctic	45.083995	-93.521909
15B542	US (*V. cardui*)	Nearctic	34.744115	-98.531672
15B003	US (*V. cardui*)	Nearctic	38.916178	-98.508965
15R082	US (*V. annabella*)	Nearctic	24.008116	-104.528997
15R092	US (*V. annabella*)	Nearctic	20.457003	-100.009924
15R119	US (*V. annabella*)	Nearctic	21.918357	-101.373286

Supplementary Table 2. Sample-specific details of the *ipyrad* assembly, reads before filtering, and loci remaining after filtering.

Sample ID	Reads before Filtering	Loci after Filtering
04G134	1005051	2755
08H295	1344081	6025
08H389	874760	5766
08H490	1498302	5977
08H640	1250840	5885
08H927	968457	5679
08L489	1519497	5988
08M443	1386673	6001
08P687	1271831	5811
08R278	1786607	6054
08R323	1587338	6029
09X874	1233483	5922
10B686	1968127	6075
11D114	1665629	6007
11E103	996666	5621
11F719	1212875	5766
11F924	1690776	6043
12L581	912176	5664
12M102	1687715	6033
12N243	1490858	5991
12P542	1454440	5969
13T444	1258683	5912
13U246	1248078	5943
14C075	1567880	6010
14C290	1724297	6004
14C924	1363395	5989
14D218	1252864	5890
14E990	1203245	5853
14F318	1374279	5931
14G912	1298483	5840
14H461	1465923	5958
14I786	1483513	5948
14V235	1073027	5862
14W965	1304164	5904
15B183	1724834	6058
15B535	1491662	5888
15B536	1493522	5846
15B589	1395550	5869
15B590	1432592	5851
15B591	1351011	5822
15B598	1108498	5790
15B641	1624115	5633
15B667	1339485	5654
15B668	1246437	5687
15B682	833936	5147
15B732	1528540	5715
15B802	1233318	5620

MOLECULAR ECOLOGY

15B803	1171253	5621
15B804	964078	5466
15B805	1295410	5664
15B806	1507144	5724
15B811	1705826	5819
15F232	1425196	5967
15F862	1447001	5862
15L546	1075818	5805
16B298	1589706	5954
16B877	1087228	5770
16B888	1196448	5857
16B889	1237145	5844
16B891	1171526	5785
16B892	1279117	5910
16E411	1526056	5891
16E439	956469	5666
16E491	1315701	5850
16E548	1234176	5763
16I034	1505964	6032
16I365	1180732	5818
16I757	1379832	5868
16I905	1276300	5919
16L312	818674	5270

Supplementary Table 3. Results of d3 tests. Introgression was tested between *V. atalanta* and its sympatric *Vanessa* species, as well as the allopatric *V. indica.* Three replicates for each test were run independently using independent

MOLECULAR ECOLOGY

sample sets for each taxon comparison. The table indicates the number of loci included in the assemblies, the d3 values obtained for each test, and the associated *p*-values. Significant introgression was detected only between *V. atalanta* and *V.indica/V.vulcania*. P-values < 0.05 are highlighted in bold. Columns 1-3 indicate the species (and region) of origin of each sample. Columns 4-6 indicate the sample IDs used in each case, corresponding to the species (and regions) indicated in columns 1-3 respectively.

Species A (Realm)	Species B (Realm)	Species C (Realm)	ID Sp. A	ID Sp. B	ID Sp. C	Number Loci	d3	p-value
V. atalanta (Palearctic)	*V. atalanta* (Nearctic)	*V. indica* (Asia)	10B686	15B183	16E165	2049	0.016	**2.17e-4**
			08R278	15B536	16E164	1918	0.014	**1.92e-3**
			14C290	15B806	16E178	1659	0.016	**1.35e-3**
V. atalanta (Palearctic)	*V. atalanta* (Nearctic)	*V. vulcania* (Macaronesia)	10B686	15B183	16L310	1754	0.011	**0.015**
			08R278	15B536	16L311	1594	0.013	**0.006**
			14C290	15B806	16L310	1552	0.012	**0.006**
V. atalanta (Nearctic)	*V. atalanta* (Palearctic)	*V. cardui* (Palearctic)	15B183	10B686	09X202	1000	-0.003	0.133
			15B536	08R278	09V399	1063	0.004	0.507
			15B806	14C290	14H393	853	-0.003	0.647
V. atalanta (Palearctic)	*V. atalanta* (Nearctic)	*V. virginiensis* (Nearctic)	10B686	16E491	16E483	1023	0.007	0.245
			08R278	15B536	15B537	1005	0.009	0.149
			14C290	16B877	16B895	1129	0.001	0.774
V. atalanta (Palearctic)	*V. atalanta* (Nearctic)	*V. cardui* (Nearctic)	10B686	15B183	15B724	1000	0.007	0.157
			08R278	15B536	15B542	883	-0.004	0.506
			14C290	15B806	15B003	918	0.005	0.421
V. atalanta (Palearctic)	*V. atalanta* (Nearctic)	*V. annabella* (Nearctic)	10B686	16B888	15R082	1069	-0.002	0.701
			08R278	16B877	15R092	792	-0.004	0.459
			14C290	16E439	15R119	872	-0.005	0.481

Supplementary Table 4. Sampling coordinates of the individuals used for the morphometrical analysis.

Sample ID	Country	Region	Latitude	Longitude
06M897	Romania	Palearctic	46.724	23.648

07D077	Romania	Palearctic	45.094	26.533
07D596	Romania	Palearctic	45.175	22.301
08H295	Spain	Palearctic	41.634	2.353
08H389	Spain	Palearctic	39.535	-0.459
08H640	Spain	Palearctic	36.199	-5.330
08H927	Spain	Palearctic	40.306	-5.806
08L489	Spain	Palearctic	42.765	0.711
08M443	Romania	Palearctic	44.062	27.649
08P687	Spain	Palearctic	42.949	-5.744
08R323	Spain	Palearctic	39.732	2.648
09X874	Italy	Palearctic	40.049	9.300
10B686	France	Palearctic	44.032	6.172
11D114	Italy	Palearctic	36.785	11.981
11E103	Malta	Palearctic	35.859	14.400
11F719	Morocco	Palearctic	34.089	-4.183
11F924	Morocco	Palearctic	33.542	-5.115
12L581	Portugal	Palearctic	37.025	-8.939
12N243	Tunisia	Palearctic	36.294	9.811
12N594	Algeria	Palearctic	35.673	3.928
12P542	France	Palearctic	44.013	3.849
12Q788	Italy	Palearctic	39.930	16.170
13T444	Tunisia	Palearctic	36.726	8.705
13U246	Italy	Palearctic	46.193	12.351
14C075	Sweden	Palearctic	59.749	18.656
14C290	Greece	Palearctic	41.293	24.054
14C924	Bulgaria	Palearctic	41.764	23.156
14D218	Austria	Palearctic	47.283	11.884
14E845	Serbia	Palearctic	44.361	21.892
14E990	Serbia	Palearctic	44.122	20.015
14G912	Greece	Palearctic	40.320	21.649
14H461	Greece	Palearctic	37.527	22.346
14I786	Slovakia	Palearctic	49.239	20.101
14L115	Algeria	Palearctic	35.300	1.130
14W208	UK	Palearctic	51.300	-1.020
14W965	UK	Palearctic	50.570	-3.900
15B535	USA	Nearctic	34.744	-98.532
15B536	USA	Nearctic	34.744	-98.532
15B565	USA	Nearctic	38.312	-95.964
15B590	USA	Nearctic	38.899	-98.520
15B591	USA	Nearctic	38.899	-98.520
15B641	USA	Nearctic	45.255	-122.904
15B667	USA	Nearctic	42.684	-103.518
15B668	USA	Nearctic	42.684	-103.518
15B682	USA	Nearctic	45.283	-95.967
15B732	USA	Nearctic	43.092	-89.336
15B802	USA	Nearctic	40.768	-73.974
15B804	USA	Nearctic	40.731	-74.000
15B805	USA	Nearctic	40.697	-73.999
15B806	USA	Nearctic	40.719	-74.016

MOLECULAR ECOLOGY

15F862	France	Palearctic	46.433	-1.027
15L546	Italy	Palearctic	43.993	8.153
16B298	France	Palearctic	45.920	5.392
16B877	Mexico	Nearctic	25.554	-100.268
16B888	Mexico	Nearctic	25.154	-99.826
16B891	Mexico	Nearctic	25.154	-99.826
16E439	Mexico	Nearctic	-	-
16E491	USA	Nearctic	35.639	-77.361
16E548	Germany	Palearctic	50.491	11.556
16E555	Algeria	Palearctic	-	-
16I034	Poland	Palearctic	52.819	14.235
16I365	Poland	Palearctic	51.406	23.124
16I757	Poland	Palearctic	53.455	20.736
16I905	Slovakia	Palearctic	48.900	19.738
16L312	Spain	Palearctic	28.391	-16.562

Supplementary Table 5. Fastsimcoal2 parameter estimates, likelihoods, AIC values and model weight for the demographic scenarios tested. The models are ranked by complexity, with M1 being the simplest, null model where

no populations experience a bottleneck, and M4 being the most complex (both populations experience a bottleneck). The best-fitting model, M4, which provided the lowest AIC value and the highest weight, is highligted in bold and marked with asterisks. The column "Par" indicates the number of parameters in the model, while **Δ**L represents the difference between the observed likelihood of the best run for each model and the maximum expected likelihood of the data. The column "AIC25" shows the AIC scores with a penalty of 25 points per parameter, and "ΔAIC25"s indicates the increase in AIC relative to the best model (M4).

**Model**	**Par**	**ΔL**	**AIC25**	**ΔAIC25**	**Weight**
M1	4	889.2	354998.2	2157.6	0
M1-migration	6	870.1	354860.3	2019.7	0
M1-one-migration	8	390.5	353001.9	161.3	9.43e-36
M2	4	2817.7	364179.6	11339.0	0
M2-one-migration	8	367.4	352895.4	54.8	1.26e-12
M3	4	393.1	352914.0	73.4	1.15e-16
M3-one-migration	8	392.9	353013.1	172.5	3.48e-38
**M4**	**6**	**366.4**	**352840.6**	**0.0**	**1*******
M4-one-migration	10	366.5	352941.1	100.5	1.50e-22

MOLECULAR ECOLOGY

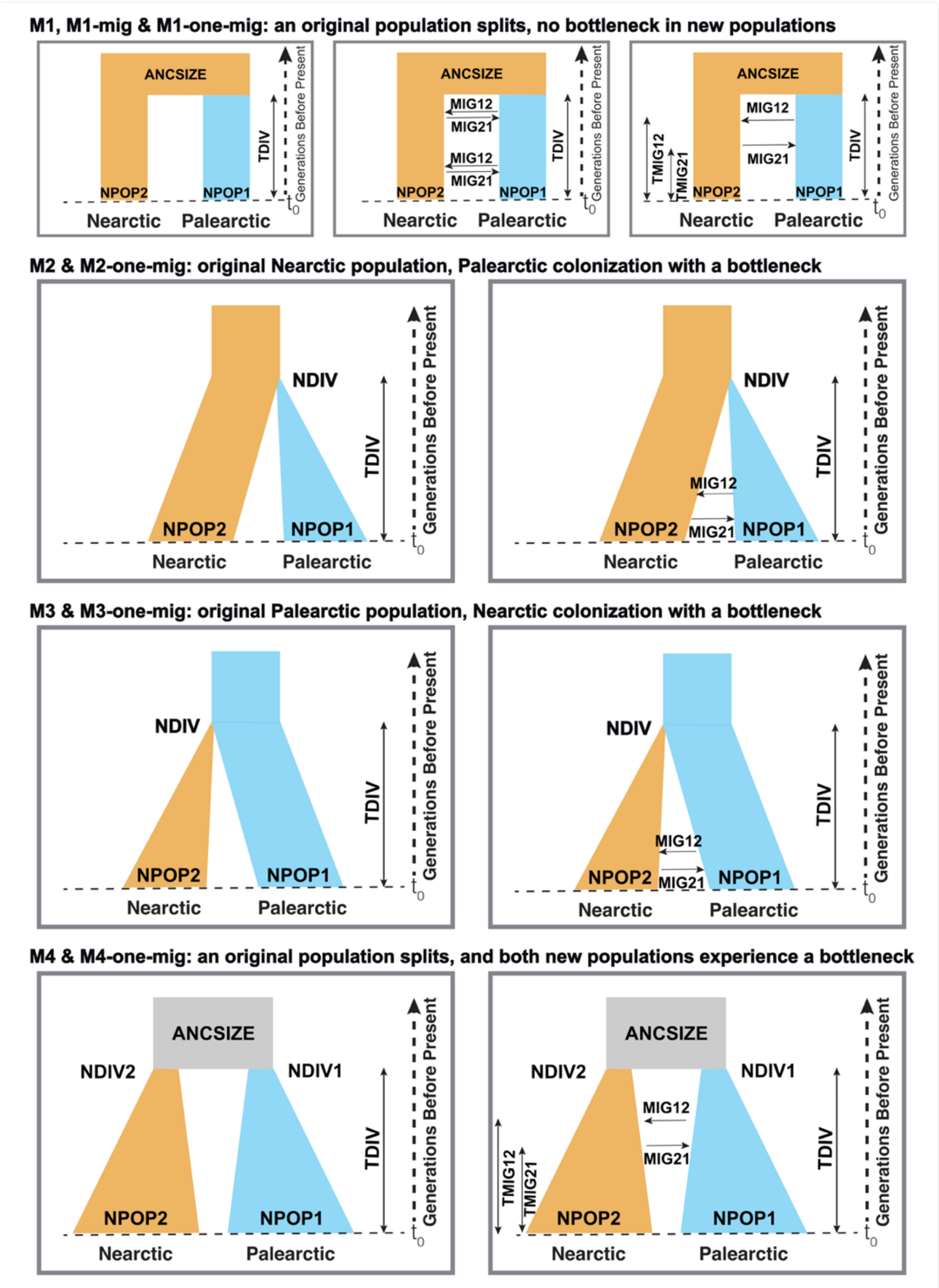

Supplementary Figure 1. Demographic models tested with *fastsimcoal2*. Orange sections of the demographic history indicate occurrence in the Nearctic, where the species was originally restricted. The blue section indicates the appearance of the Palearctic *V. atalanta* population. N_e is indicated in terms of haploid genomes, and time in generations BP, with more recent times on the lower end.

MOLECULAR ECOLOGY

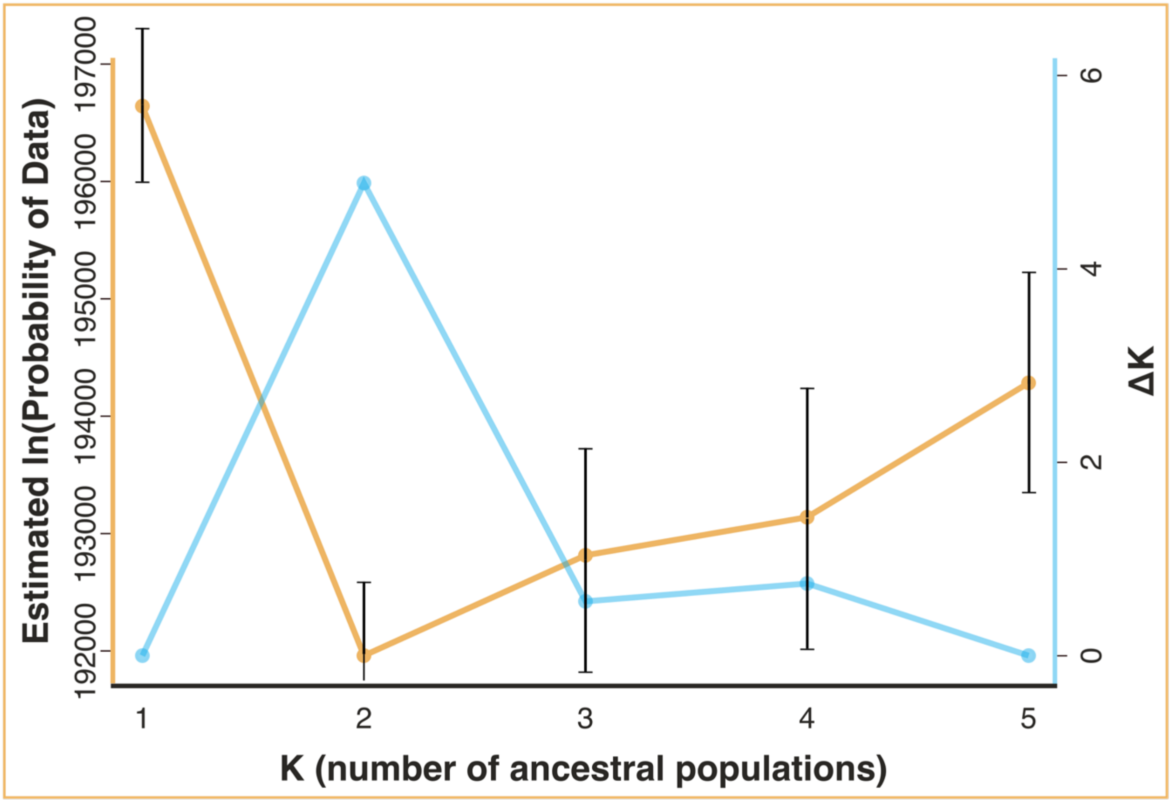

Supplementary Figure 2. Results of clustering analyses with STRUCTURE. Both the log(likelihood) of the data and ΔK are shown. Lower values of log(likelihood) and higher ΔK indicate the number of clusters that provides the best fit for the data.

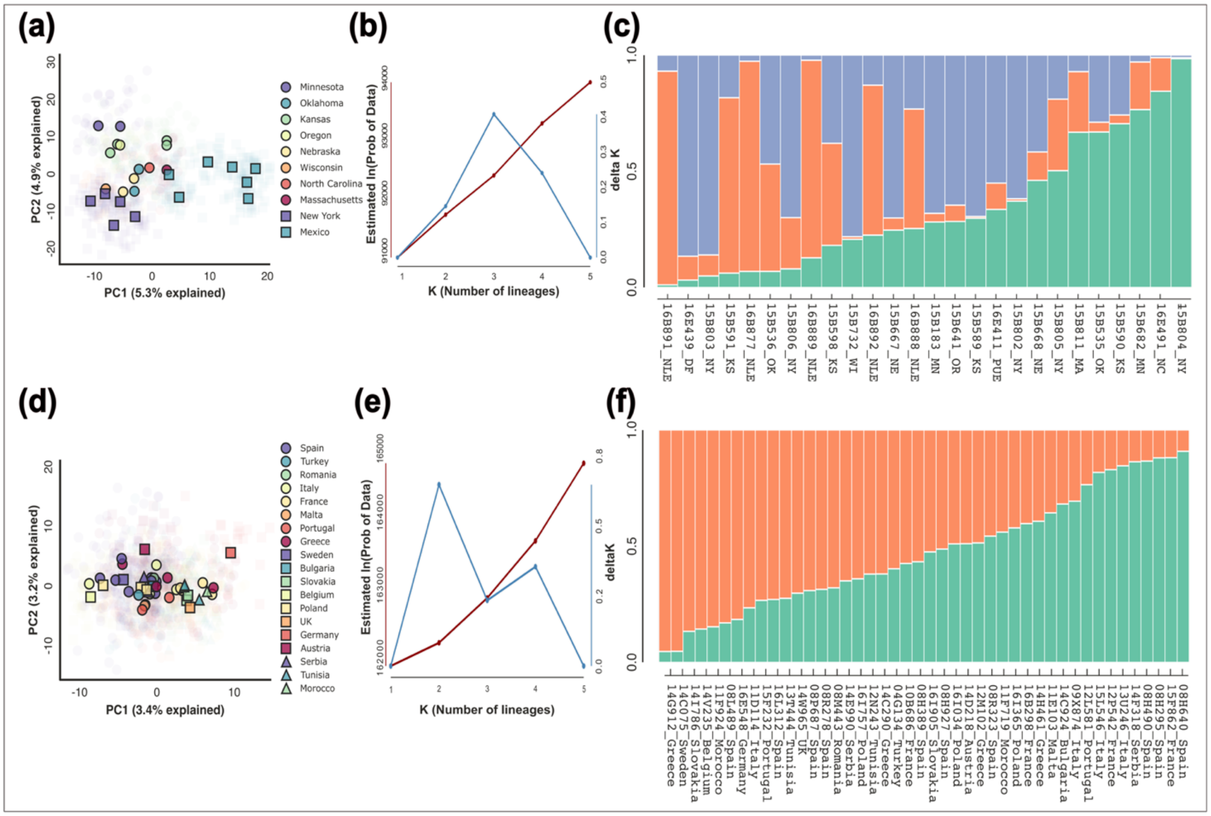

Supplementary Figure 3. Patterns of intra-continental structure in *V. atalanta* in the Nearctic (a-c) and the Palearctic (d-f). (a) PCA for the Nearctic specimens. Samples are colored by state/country of origin. **(b)** ΔK (blue) and likelihood of the data (red) for the STRUCTURE analyses of the Nearctic samples. ΔK supports the existence of K = 3 lineages. **(c)** STRUCTURE plot for the Nearctic samples. Each column represents an individual, and the name includes the sample ID followed by the two- (USA) or three-letter (Mexico) abbreviations of the state of origin. **(d)** PCA for the Palearctic specimens. Samples are colored by country of origin. **(e)** K (blue) and likelihood of the data (red) for the STRUCTURE analyses of the Palearctic samples. ΔK supports the existence of K = 2 lineages. **(f)** STRUCTURE plot for the Palearctic samples. Each column represents an individual, and the name includes the sample ID followed by the country of origin.

MOLECULAR ECOLOGY

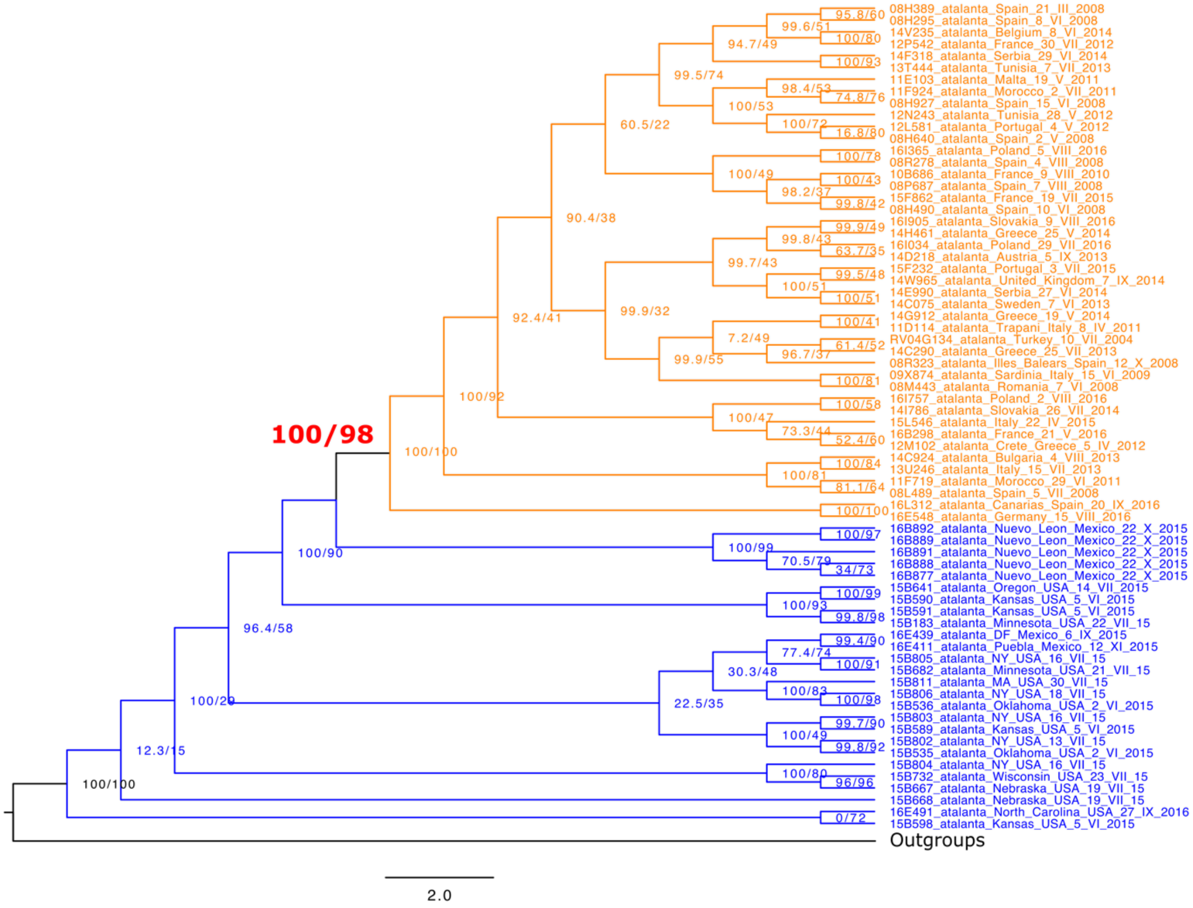

Supplementary Figure 4. Phylogeny of *Vanessa atalanta*. Cladogram obtained from RAD data of 70 *V. atalanta* samples using IQ-TREE v2.3.2. Two *V. cardui* individuals were used as outgroup, and are shown here collapsed into a single branch. The node that represents the split of the monophyletic Palearctic *V. atalanta* samples (indicated as well in Figure 1b) is shown in red and bold. Branch support values are shown for the SH-aLRT/ultra-fast bootstrap.

MOLECULAR ECOLOGY

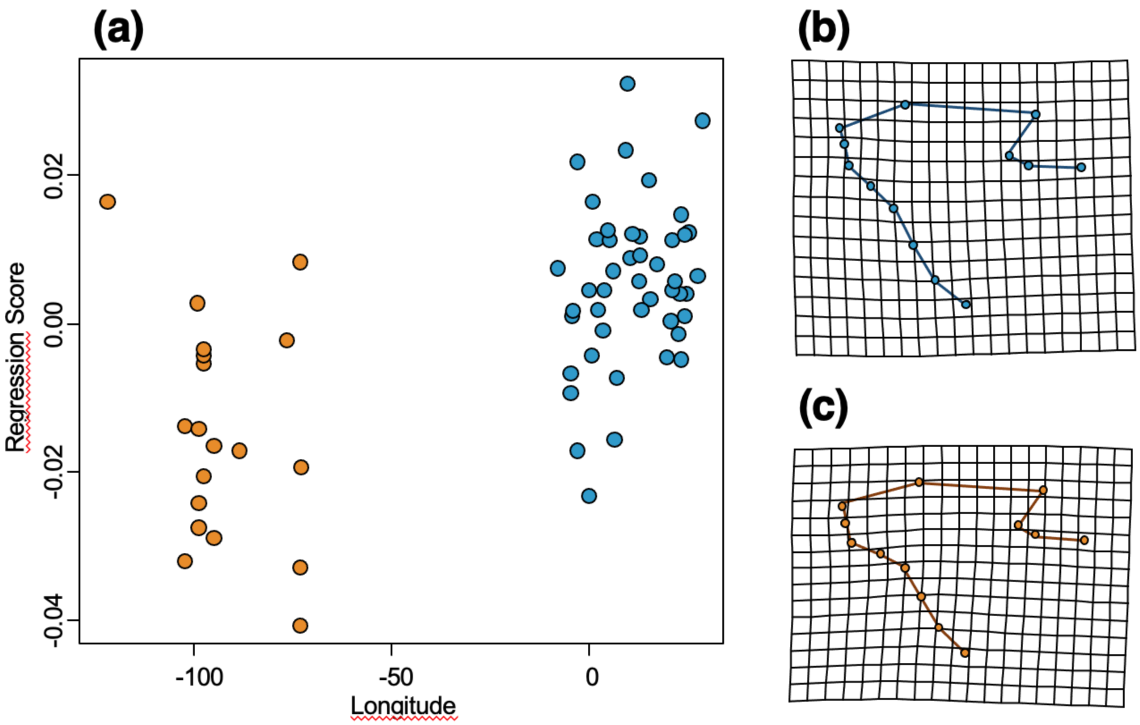

Supplementary Figure 5. Sample longitude is associated with shape variation in *V. atalanta*. (a) GPA regression score is significantly associated with the longitude of origin of the samples used in the morphometric analysis. **(b)** Thin plate spline for the Palearctic samples. **(c)** Thin plate spline for the Nearctic samples.

MOLECULAR ECOLOGY

Appendix 1 – *fastsimcoal2* model determination files

M1.tpl

```
//Number of population samples (demes)
2 demes (populations) will be simulated, corresponding to NA and
Palearctic
//Population effective sizes (number of genes)
NPOP1 Population size of Palearctic
NPOP2 Population size of North America
//Sample sizes (can include sampling time and inbreeding level
separated by space, but since we assume constant size it is not
needed)
64
38
//Growth rates : constant population size
0
0
//Number of migration matrices : 0 implies no migration between demes
0
//historical event: time, source, sink, migrants, new size, new growth
rate, migr. matrix
1 historical events
TDIV 1 0 1 ANCSIZE 0 0 absoluteResize
//Number of independent loci [chromosome]
1 0 //1 chromosomes with similar structure
//Per chromosome: Number of linkage blocks
1
//per Block: data type, num loci, rec. rate and mut rate + optional
parameters
FREQ 1 0 2.9e-9 OUTEXP
```

M1.est

```
// Priors and rules file
// *********************

[PARAMETERS]
//#isInt? #name #dist. #min #max
//all N are in number of haploid individuals

1       ANCSIZE unif    2000000 10000000        output
1       NPOP1   unif    1116314 2114575 output
1       NPOP2   unif    3061271 8158885 output
1       TDIV    unif    2000    40000   output


[COMPLEX PARAMETERS]
```

MOLECULAR ECOLOGY

M1-mig.tpl

```
//Number of population samples (demes)
2 demes (populations) will be simulated, corresponding to NA and
Palearctic
//Population effective sizes (number of genes)
NPOP1 Population size of Palearctic
NPOP2 Population size of North America
//Sample sizes (can include sampling time and inbreeding level
separated by space, but since we assume constant size it is not
needed)
64
38
//Growth rates : constant population size
0
0
//Number of migration matrices : 0 implies no migration between demes
2
//Migration matrix 0
0       MIG12
MIG21   0
//Migration matrix 1
0       0
0       0
//historical event: time, source, sink, migrants, new size, new growth
rate, migr. matrix
1 historical events
TDIV 1 0 1 ANCSIZE 0 1 absoluteResize
//Number of independent loci [chromosome]
1 0 //1 chromosomes with similar structure
//Per chromosome: Number of linkage blocks
1
//per Block: data type, num loci, rec. rate and mut rate + optional
parameters
FREQ 1 0 2.9e-9 OUTEXP
```

MOLECULAR ECOLOGY

M1-mig.est

```
// Priors and rules file
// *********************

[PARAMETERS]
//#isInt? #name #dist. #min #max
//all N are in number of haploid individuals

1       ANCSIZE unif    2000000 10000000        output
1       NPOP1   unif    1116314 2114575 output
1       NPOP2   unif    3061271 8158885 output
1       TDIV    unif    2000    40000   output
0       MIG12   logunif 1e-10   1e-4    output
0       MIG21   logunif 1e-10   1e-4    output

[COMPLEX PARAMETERS]
```

MOLECULAR ECOLOGY

M1-one-mig.tpl

```
//Number of population samples (demes)
2 demes (populations) will be simulated, corresponding to NA and
Palearctic
//Population effective sizes (number of genes)
NPOP1 Population size of Palearctic
NPOP2 Population size of North America
//Sample sizes (can include sampling time and inbreeding level
separated by space, but since we assume constant size it is not
needed)
64
38
//Growth rates : constant population size
0
0
//Number of migration matrices : 0 implies no migration between demes
0
//historical event: time, source, sink, migrants, new size, new growth
rate, migr. matrix
3 historical events
TDIV 1 0 1 ANCSIZE 0 0 absoluteResize
TMIG12 0 1 M12 1 0 0
TMIG21 1 0 M21 1 0 0
//Number of independent loci [chromosome]
1 0 //1 chromosomes with similar structure
//Per chromosome: Number of linkage blocks
1
//per Block: data type, num loci, rec. rate and mut rate + optional
parameters
FREQ 1 0 2.9e-9 OUTEXP
M1-one-mig.est
```

MOLECULAR ECOLOGY

M1-one-mig.est

```
// Priors and rules file
// *********************

[PARAMETERS]
//#isInt? #name #dist. #min #max
//all N are in number of haploid individuals

1       ANCSIZE unif    1000000 10000000        output
1       NPOP1   unif    1116314 2114575 output
1       NPOP2   unif    3061271 8158885 output
1       TDIV    unif    2000    40000   output
1       TMIG12  unif    100     TDIV    output  paramInRange
1       TMIG21  unif    100     TDIV    output  paramInRange
0       M12     logunif 1e-10   1e-4    output
0       M21     logunif 1e-10   1e-4    output

[COMPLEX PARAMETERS]
```

MOLECULAR ECOLOGY

M2.tpl

```
//Number of population samples (demes)
2 demes (populations) will be simulated, corresponding to NA and
Palearctic
//Population effective sizes (number of genes)
NPOP1 Population size of Palearctic
NPOP2 Population size of North America
//Sample sizes (can include sampling time and inbreeding level
separated by space, but since we assume constant size it is not
needed)
64
38
//Growth rates : constant population size
R1
0
//Number of migration matrices : 0 implies no migration between demes
0
//historical event: time, source, sink, migrants, new size, new growth
rate, migr. matrix
1 historical events
TDIV 0 1 1 1 0 0
//Number of independent loci [chromosome]
1 0
//Per chromosome: Number of linkage blocks
1
//per Block: data type, num loci, rec. rate and mut rate + optional
parameters
FREQ 1 0 2.9e-9 OUTEXP
```

MOLECULAR ECOLOGY

M2.est

```
// Priors and rules file
// *********************

[PARAMETERS]
//#isInt? #name #dist. #min #max
//all N are in number of haploid individuals

1       NPOP1   unif    1116314 2114575 output
1       NPOP2   unif    3061271 8158885 output
1       NDIV    unif    100     1000000 output
1       TDIV    unif    2000    40000   output

[COMPLEX PARAMETERS]
0       RATIO_OOA_EA = NDIV/NPOP1       hide
0       RTEA = log(RATIO_OOA_EA)        hide
0       R1 = RTEA/TDIV  hide
```

MOLECULAR ECOLOGY

M2-one-mig.tpl

```
//Number of population samples (demes)
2 demes (populations) will be simulated, corresponding to NA and
Palearctic
//Population effective sizes (number of genes)
NPOP1 Population size of Palearctic
NPOP2 Population size of North America
//Sample sizes (can include sampling time and inbreeding level
separated by space, but since we assume constant size it is not
needed)
64
38
//Growth rates : constant population size
R1
0
//Number of migration matrices : 0 implies no migration between demes
0
//historical event: time, source, sink, migrants, new size, new growth
rate, migr. matrix
3 historical events
TDIV 0 1 1 1 0 0
TMIG12 0 1 M12 1 R1 0
TMIG21 1 0 M21 1 R1 0
//Number of independent loci [chromosome]
1 0
//Per chromosome: Number of linkage blocks
1
//per Block: data type, num loci, rec. rate and mut rate + optional
parameters
FREQ 1 0 2.9e-9 OUTEXP
```

MOLECULAR ECOLOGY

M2-one-mig.est

```
// Priors and rules file
// *********************

[PARAMETERS]
//#isInt? #name #dist. #min #max
//all N are in number of haploid individuals

1       NPOP1   unif    1116314 2114575 output
1       NPOP2   unif    3061271 8158885 output
1       NDIV    unif    100     1000000 output
1       TDIV    unif    2000    40000   output
1       TMIG12  unif    100     TDIV    output  paramInRange
1       TMIG21  unif    100     TDIV    output  paramInRange
0       M12     logunif 1e-10   1e-4    output
0       M21     logunif 1e-10   1e-4    output

[COMPLEX PARAMETERS]
0       RATIO_OOA_EA = NDIV/NPOP1       hide
0       RTEA = log(RATIO_OOA_EA)        hide
0       R1 = RTEA/TDIV  hide
```

MOLECULAR ECOLOGY

M3.tpl

```
//Number of population samples (demes)
2 demes (populations) will be simulated, corresponding to NA and
Palearctic
//Population effective sizes (number of genes)
NPOP1 Population size of Palearctic
NPOP2 Population size of North America
//Sample sizes (can include sampling time and inbreeding level
separated by space, but since we assume constant size it is not
needed)
64
38
//Growth rates : constant population size
0
R1
//Number of migration matrices : 0 implies no migration between demes
0
//historical event: time, source, sink, migrants, new size, new growth
rate, migr. matrix
1 historical events
TDIV 1 0 1 1 0 0
//Number of independent loci [chromosome]
1 0
//Per chromosome: Number of linkage blocks
1
//per Block: data type, num loci, rec. rate and mut rate + optional
parameters
FREQ 1 0 2.9e-9 OUTEXP
```

MOLECULAR ECOLOGY

M3.est

```
// Priors and rules file
// *********************

[PARAMETERS]
//#isInt? #name #dist. #min #max
//all N are in number of haploid individuals

1       NPOP1   unif    1116314 2114575 output
1       NPOP2   unif    3061271 8158885 output
1       NDIV    unif    100     1000000 output
1       TDIV    unif    2000    40000   output

[COMPLEX PARAMETERS]
0       RATIO_OOA_EA = NDIV/NPOP2       hide
0       RTEA = log(RATIO_OOA_EA)        hide
0       R1 = RTEA/TDIV  hide
```

MOLECULAR ECOLOGY

M3-one-mig.tpl

```
//Number of population samples (demes)
2 demes (populations) will be simulated, corresponding to NA and
Palearctic
//Population effective sizes (number of genes)
NPOP1 Population size of Palearctic
NPOP2 Population size of North America
//Sample sizes (can include sampling time and inbreeding level
separated by space, but since we assume constant size it is not
needed)
64
38
//Growth rates : constant population size
0
R1
//Number of migration matrices : 0 implies no migration between demes
0
//historical event: time, source, sink, migrants, new size, new growth
rate, migr. matrix
3 historical events
TDIV 1 0 1 1 0 0
TMIG12 0 1 M12 1 0 0
TMIG21 1 0 M21 1 0 0
//Number of independent loci [chromosome]
1 0
//Per chromosome: Number of linkage blocks
1
//per Block: data type, num loci, rec. rate and mut rate + optional
parameters
FREQ 1 0 2.9e-9 OUTEXP
```

M3-one-mig.est

```
// Priors and rules file
// *********************

[PARAMETERS]
//#isInt? #name #dist. #min #max
//all N are in number of haploid individuals

1       NPOP1   unif    1116314 2114575 output
1       NPOP2   unif    3061271 8158885 output
1       NDIV    unif    100     1000000 output
1       TDIV    unif    2000    40000   output
1       TMIG12  unif    100     TDIV    output  paramInRange
1       TMIG21  unif    100     TDIV    output  paramInRange
0       M12     logunif 1e-10   1e-4    output
0       M21     logunif 1e-10   1e-4    output

[COMPLEX PARAMETERS]
0       RATIO_OOA_EA = NDIV/NPOP2       hide
0       RTEA = log(RATIO_OOA_EA)        hide
0       R1 = RTEA/TDIV  hide
```

MOLECULAR ECOLOGY

M4.tpl

```
//Number of population samples (demes)
2 demes (populations) will be simulated, corresponding to NA and
Palearctic
//Population effective sizes (number of genes)
NPOP1 Population size of Palearctic
NPOP2 Population size of North America
//Sample sizes (can include sampling time and inbreeding level
separated by space, but since we assume constant size it is not
needed)
64
38
//Growth rates : constant population size
R1
R2
//Number of migration matrices : 0 implies no migration between demes
0
//historical event: time, source, sink, migrants, new size, new growth
rate, migr. matrix
2 historical events
TDIV 0 0 0 ANCSIZE 0 0 absoluteResize
TDIV 1 0 1 ANCSIZE 0 0 absoluteResize
//Number of independent loci [chromosome]
1 0
//Per chromosome: Number of linkage blocks
1
//per Block: data type, num loci, rec. rate and mut rate + optional
parameters
FREQ 1 0 2.9e-9 OUTEXP
```

M4.est

```
// Priors and rules file
// *********************

[PARAMETERS]
//#isInt? #name #dist. #min #max
//all N are in number of haploid individuals

1       ANCSIZE unif    1000000 10000000        output
1       NPOP1   unif    1116314 2114575 output
1       NPOP2   unif    3061271 8158885 output
1       NDIV1   unif    100     1000000 output
1       NDIV2   unif    100     1000000 output
1       TDIV    unif    2000    40000   output

[COMPLEX PARAMETERS]
0       RATIO_OOA_EA1 = NDIV1/NPOP1     hide
0       RTEA1 = log(RATIO_OOA_EA1)      hide
0       R1 = RTEA1/TDIV hide
0       RATIO_OOA_EA2 = NDIV2/NPOP2      hide
0       RTEA2 = log(RATIO_OOA_EA2)      hide
0       R2 = RTEA2/TDIV hide
```

MOLECULAR ECOLOGY

M4-one-mig.tpl

```
//Number of population samples (demes)
2 demes (populations) will be simulated, corresponding to NA and
Palearctic
//Population effective sizes (number of genes)
NPOP1 Population size of Palearctic
NPOP2 Population size of North America
//Sample sizes (can include sampling time and inbreeding level
separated by space, but since we assume constant size it is not
needed)
64
38
//Growth rates : constant population size
R1
R2
//Number of migration matrices : 0 implies no migration between demes
0
//historical event: time, source, sink, migrants, new size, new growth
rate, migr. matrix
3 historical events
TDIV 1 0 1 ANCSIZE 0 0 absoluteResize
TMIG12 0 1 M12 1 0 0
TMIG21 1 0 M21 1 0 0
//Number of independent loci [chromosome]
1 0
//Per chromosome: Number of linkage blocks
1
//per Block: data type, num loci, rec. rate and mut rate + optional
parameters
FREQ 1 0 2.9e-9 OUTEXP
```

MOLECULAR ECOLOGY

M4-one-mig.est

```
// Priors and rules file
// *********************

[PARAMETERS]
//#isInt? #name #dist. #min #max
//all N are in number of haploid individuals

1       ANCSIZE unif    1000000 10000000        output
1       NPOP1   unif    1116314 2114575 output
1       NPOP2   unif    3061271 8158885 output
1       NDIV1   unif    100     1000000 output
1       NDIV2   unif    100     1000000 output
1       TDIV    unif    2000    40000   output
1       TMIG12  unif    100     TDIV    output  paramInRange
1       TMIG21  unif    100     TDIV    output  paramInRange
0       M12     logunif 1e-10   1e-4    output
0       M21     logunif 1e-10   1e-4    output

[COMPLEX PARAMETERS]
0       RATIO_OOA_EA1 = NDIV1/NPOP1     hide
0       RTEA1 = log(RATIO_OOA_EA1)      hide
0       R1 = RTEA1/TDIV hide
0       RATIO_OOA_EA2 = NDIV2/NPOP2      hide
0       RTEA2 = log(RATIO_OOA_EA2)      hide
0       R2 = RTEA2/TDIV hide
```
